# A Metabolomics-Driven Integrated Machine Learning Framework for Early Prediction of Recurrent Acute Pancreatitis

**DOI:** 10.3390/metabo16080575

**Published:** 2026-08-15

**Authors:** Qing Huang, Xiaoyan Shen, Ying Li, Haoxi Wu, Xiangxiang Huang, Guangming Li, Di Wang

**Affiliations:** 1 School of Life and Health Technology, Dongguan University of Technology, Dongguan 523808, China; 241115308@dgut.edu.cn (Q.H.);; 2 School of Computer Science and Technology, Dongguan University of Technology, Dongguan 523808, Chinaligm@dgut.edu.cn (G.L.); 3 The First School of Clinical Medicine, Guangdong Medical University, Dongguan 523808, China; leeyin9@163.com

**Keywords:** recurrent acute pancreatitis, metabolomics, machine learning, adaptive feature selection, early risk prediction

## Abstract

**Background/Objective**: Recurrent acute pancreatitis (RAP) can progress to chronic pancreatitis and ultimately pancreatic ductal adenocarcinoma; therefore, early identification of patients at high recurrence risk is clinically important. However, the high dimensionality, inherent variability, and susceptibility to outliers of metabolomic data pose challenges for reliable biomarker identification. **Methods**: Here, we propose an integrated framework that combines Isolation Forest-based outlier handling to systematically assess the influence of aberrant samples, a dual-pathway adaptive Lasso feature selection strategy incorporating stability-oriented and sparsity-oriented pathways, and statistical significance filtering to reduce false positives. **Results**: When applied to a cohort of 63 patients, Random Forest (RF) achieved the highest mean outer-fold area under the receiver operating characteristic curve (AUC) among the five classifiers evaluated (0.933 ± 0.133). In an exploratory modality analysis incorporating 11 prespecified baseline clinical covariates, the clinical-only, metabolomics-only, and combined clinical–metabolomic RF models achieved mean outer-fold AUCs of 0.712 ± 0.265, 0.933 ± 0.133, and 0.986 ± 0.029, respectively. Although the combined model showed the highest numerical AUC, it did not significantly outperform the metabolomics-only model based on pooled out-of-fold predictions. Notably, in this small-sample setting, the dual-pathway selection strategy recurrently identified orotic acid as a candidate metabolite with the most consistent fold-wise statistical support across outer training partitions. Re-execution of the workflow on a public gastric cancer metabolomics dataset demonstrated its technical portability to a distinct classification task. **Conclusions**: Together, these findings provide a framework that distinguishes internal predictive performance and feature stability from biochemical validation, offering a reproducible basis for prioritizing RAP-associated metabolic signals and guiding future multicenter targeted validation studies.

## 1. Introduction

Acute pancreatitis (AP) is among the most common pancreatic diseases, with approximately 2.75 million new cases reported globally in 2021 [1,2]. Up to 20% of patients may develop recurrent acute pancreatitis (RAP) after an index episode [3]. Recurrent episodes impose a substantial burden through repeated hospitalizations and are associated with progression to chronic pancreatitis (CP) and an increased long-term risk of pancreatic ductal adenocarcinoma (PDAC) [4,5]. Severe episodes can result in multiple-organ failure and death [6]. Therefore, early identification of patients at high risk of recurrence could support risk-adapted follow-up and preventive management.

Oxidative stress drives lipid peroxidation, generating reactive aldehydes. In pancreatitis models, elevated lipid-peroxidation markers are linked to pancreatic injury and inflammation [7,8,9], supporting metabolomic profiling to explore recurrence-related molecular patterns.

Metabolomics, as a high-throughput omics technology, can reveal potential associations between metabolite levels and physiological or pathological states by profiling endogenous small molecules in biological systems [10,11]. It has demonstrated great promise in uncovering early metabolic dysregulation in complex diseases and in identifying diagnostic and prognostic biomarkers [12,13,14]. By comprehensively analyzing blood or urine metabolites, metabolomics provides a powerful platform for mapping disease-specific perturbations in biochemical pathways, including those governing lipid metabolism and redox balance [15,16,17]. The Human Metabolome Database (HMDB) and related pathway maps have offered fundamental tools for auxiliary diagnosis in AP and associated disorders [18]. In pancreatic diseases, metabolomic studies have frequently linked altered lipid metabolites, aromatic amino acids, and bile acids with disease severity [19], while work on pancreatic cancer has identified novel metabolite biomarkers with superior accuracy over traditional markers [20].

However, metabolomics data are inherently high-dimensional, highly heterogeneous, and characterized by complex inter-metabolite correlations, which poses significant challenges for the extraction of biologically meaningful features [21,22]. Conventional statistical approaches such as principal component analysis (PCA) and partial least squares discriminant analysis (PLS-DA) are often limited in capturing nonlinear relationships and are prone to overfitting in high-dimensional settings [23,24].

Machine learning methods have been increasingly applied to construct intelligent diagnostic and predictive models for AP. For instance, an XGBoost-based clinical prediction model identified hypertriglyceridemia, alcohol consumption, and body mass index (BMI) as significant factors for RAP development, achieving an area under the receiver operating characteristic (ROC) curve (AUC) of 0.779 in external validation [25]. Radiomics-based models using support vector machines have also been explored for early RAP risk prediction in patients with metabolic syndrome [26]. At the molecular level, Tremblay et al. demonstrated that immune-inflammatory pathways are activated in RAP, suggesting that chronic low-grade inflammation contributes to recurrence risk [27]. However, it remains unclear whether untargeted metabolomic profiles provide reproducible internal predictive information when preprocessing, feature selection, and model tuning are contained within the resampling procedure.

To address this challenge, we propose an integrated adaptive metabolomics framework strategy for RAP prediction (IAMFS-RAP), which comprises three core components: (1) Isolation Forest-based outlier handling to systematically assess the influence of aberrant samples; (2) a dual-pathway adaptive Lasso strategy that incorporates stability-oriented and sparsity-oriented selection pathways; and (3) statistical significance filtering with false-discovery rate correction to reduce false positives. These components are embedded within a strict outer cross-validation scheme to ensure that all data-dependent decisions, including variance filtering, robust scaling, and hyperparameter tuning, are confined to the training folds. Collectively, this pipeline provides exploratory internal evidence of metabolomic patterns associated with RAP recurrence and establishes a reproducible methodological basis for future independent clinical and analytical validation. 

## 2. Materials and Methods

### 2.1. Dataset

#### 2.1.1. Study Population and Inclusion Criteria

**Figure 1:**
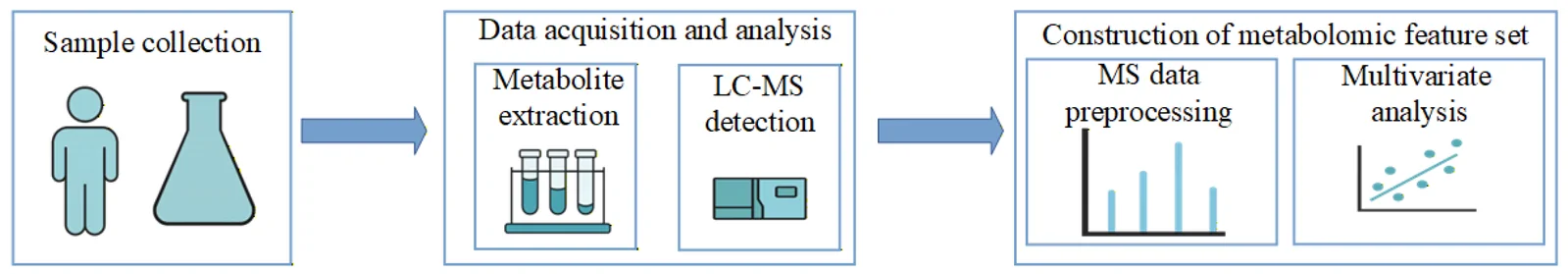
Metabolomics Data Acquisition Workflow Diagram.

Patients presenting with a first episode of AP at Shenzhen Longhua District People’s Hospital were enrolled. RAP was defined as a new episode of AP occurring within a prespecified three-month follow-up window after complete clinical resolution of the index episode and a symptom-free interval; persistence or worsening of the index episode was not classified as recurrence. Follow-up was conducted through a combination of in-person visits and telephone contacts. Suspected recurrence events were reviewed by clinicians using the revised Atlanta diagnostic framework, which required at least two of the following criteria: characteristic abdominal pain, serum amylase or lipase activity at least three times the upper limit of normal, and imaging findings consistent with AP [6]. A recurrence was recorded only after the clinician confirmed a new episode on repeat clinical assessment, laboratory testing and/or imaging. Exclusion criteria included age < 18 years at diagnosis, chronic pancreatitis, severe organ dysfunction, loss to follow-up, and missing essential clinical data. A total of 63 eligible patients were included. The study protocol was approved by the Ethics Committee of the People’s Hospital of Longhua, Shenzhen. Clinical samples were used in accordance with institutional guidelines, and informed consent was obtained from all participants. After case confirmation, non-targeted metabolomics analysis was performed using ultra-high-performance liquid chromatography-tandem mass spectrometry (UPLC-MS/MS). The overall data acquisition workflow (Figure 1) comprised sample collection, mass spectrometry detection, data preprocessing, and metabolomic feature set construction.

#### 2.1.2. Blood Sample Collection and Preparation

Blood samples were collected on the day after admission using vacuum collection tubes without anticoagulants. The samples were thawed at 4 °C and vortexed thoroughly. A 100 μL aliquot of each sample was transferred to a new tube and mixed with 100 μL of pre-cooled water and 800 μL of pre-cooled methanol/acetonitrile (1:1, *v*/*v*). After vortexing, the mixture was sonicated in an ice bath for 1 h and incubated at −20 °C for 1 h to precipitate proteins, then centrifuged at 16,000× *g* for 20 min at 4 °C. The supernatant was collected and dried using a high-speed vacuum concentrator. No targeted recovery experiment was performed to quantify extraction efficiency for individual metabolites; therefore, the effects of protein precipitation on the recovery of specific metabolites could not be quantified. For UPLC–MS/MS analysis, the dried extracts were reconstituted in 100 μL of methanol–water (1:1, *v*/*v*), centrifuged at 20,000× *g* for 20 min at 4 °C, and the final supernatant was injected. All processed samples were stored at −80 °C until analysis to minimize metabolite degradation.

Quality control (QC) samples were prepared by pooling equal aliquots of all study samples and were injected periodically throughout the analytical run to monitor analytical stability and potential batch effects.

Feature-level QC repeatability was quantified from the eight pooled-QC injections in the available QC peak-area export. For each ion feature, the coefficient of variation (CV) was calculated as the sample standard deviation divided by the mean peak area across the QC injections and expressed as a percentage. The distribution of CV values and the proportions of features below 20% and 30% were reported as descriptive repeatability benchmarks. No QC-CV threshold was applied as a filtering criterion to the primary 959-feature model input.

#### 2.1.3. UPLC–MS/MS Analysis

Reconstituted serum extracts were maintained at 4 °C in the autosampler and analyzed using a Shimadzu Nexera X2 LC-30AD ultra-high-performance liquid chromatography (UHPLC) system (Shimadzu Corporation, Kyoto, Japan) coupled to a Thermo Scientific Q Exactive Plus mass spectrometer (Thermo Fisher Scientific, Bremen, Germany). Chromatographic separation was performed on an ACQUITY UPLC HSS T3 column (2.1 × 100 mm, 1.8 μm; Waters Corporation, Milford, MA, USA) with a flow rate of 0.3 mL/min and column temperature maintained at 40 °C. The injection volume was 5 μL. The mobile phases consisted of 0.1% formic acid in water (A) and acetonitrile (B) with a 15-min gradient elution. Mass spectrometric detection was carried out in both positive and negative ion modes using a heated electrospray ionization (HESI) source supplied by Thermo Fisher Scientific (Bremen, Germany). Key parameters included spray voltages of 3.8 kV in positive-ion mode and 3.2 kV in negative-ion mode, and a capillary temperature of 320 °C. Full-scan MS data were acquired over the *m*/*z* range 70–1050 at a resolution of 70,000, followed by data-dependent MS/MS of the top 10 most abundant ions using stepped collision energies (10–30 eV).

Each serum sample was analyzed once in positive-ion mode and once in negative-ion mode, yielding two raw data files per sample. The chromatographic runtime was 15 min per injection and 30 min per sample across both ionization modes, excluding QC injections.

A pooled QC sample was prepared by combining equal aliquots of all study samples. Eight pooled-QC injections were analyzed in both positive- and negative-ion modes. Analytical stability was assessed by comparing the base-peak chromatograms of the QC injections and by PCA of the complete analytical dataset (Supplementary Figure S1).

#### 2.1.4. Metabolomic Feature Set Construction

The metabolomics service provider supplied the annotated peak-area tables used to construct the model input. According to the provider’s project report, raw LC-MS/MS data were processed using MS-DIAL for peak alignment, retention-time correction, and peak-area extraction. Putative annotations were assigned by accurate-mass matching (mass tolerance < 0.02 Da) against public and in-house spectral libraries. Ion features with >50% missing values within either outcome group were excluded during construction of the comparison table. Positive- and negative-ion data were normalized separately by total peak area and subsequently merged.

All eight reported candidate features were therefore treated as putative annotations rather than confirmed metabolite identities. The available annotation metadata comprised the alignment identifier, retention time, *m*/*z* value, adduct type, molecular formula, database identifiers and ionization mode. Feature-level mass errors, MS/MS match scores, candidate-specific QC CVs, blank-sample assessments and authentic-standard confirmations could not be reliably linked to these eight candidates in the supplied data package and are reported as unavailable in Supplementary Table S6. Candidate names are used only as database-linked labels, and no mechanistic or causal interpretation is based on these annotations.

The machine-learning analysis used a fixed matrix containing peak-area measurements for 959 ion features. Representative rows of this fixed 959-feature model-input matrix are shown in Table 1. The model-input values were not unit-variance scaled before import into the modeling workflow; the unit-variance scaling described in the provider’s report applied to the provider’s standard multivariate analyses. Because the outcome-group-informed missingness filter preceded the outer cross-validation split, its practical influence on feature eligibility was evaluated in a dedicated fold-specific audit. From the fixed 959-feature matrix onwards, all subsequent data-dependent model-development procedures, including optional outlier handling, variance filtering, RobustScaler transformation, dual-pathway feature selection, fold-specific statistical testing, and hyperparameter optimization, were fitted exclusively within the relevant training partitions.

To quantify the practical scope of the upstream outcome-group-informed missingness filter, we conducted a fold-specific feature-eligibility audit using the earliest available processed RAP/non-RAP table, which contained 961 ion features. The >50% missingness rule was reapplied independently within each of the five original outer-training partitions. Fold-specific retained feature sets were compared with the full-cohort retained set using feature counts and Jaccard similarity, and the eligibility of the eight reported candidate features was examined. This audit assessed feature-space sensitivity rather than classifier performance.

### 2.2. Methods

**Figure 2:**
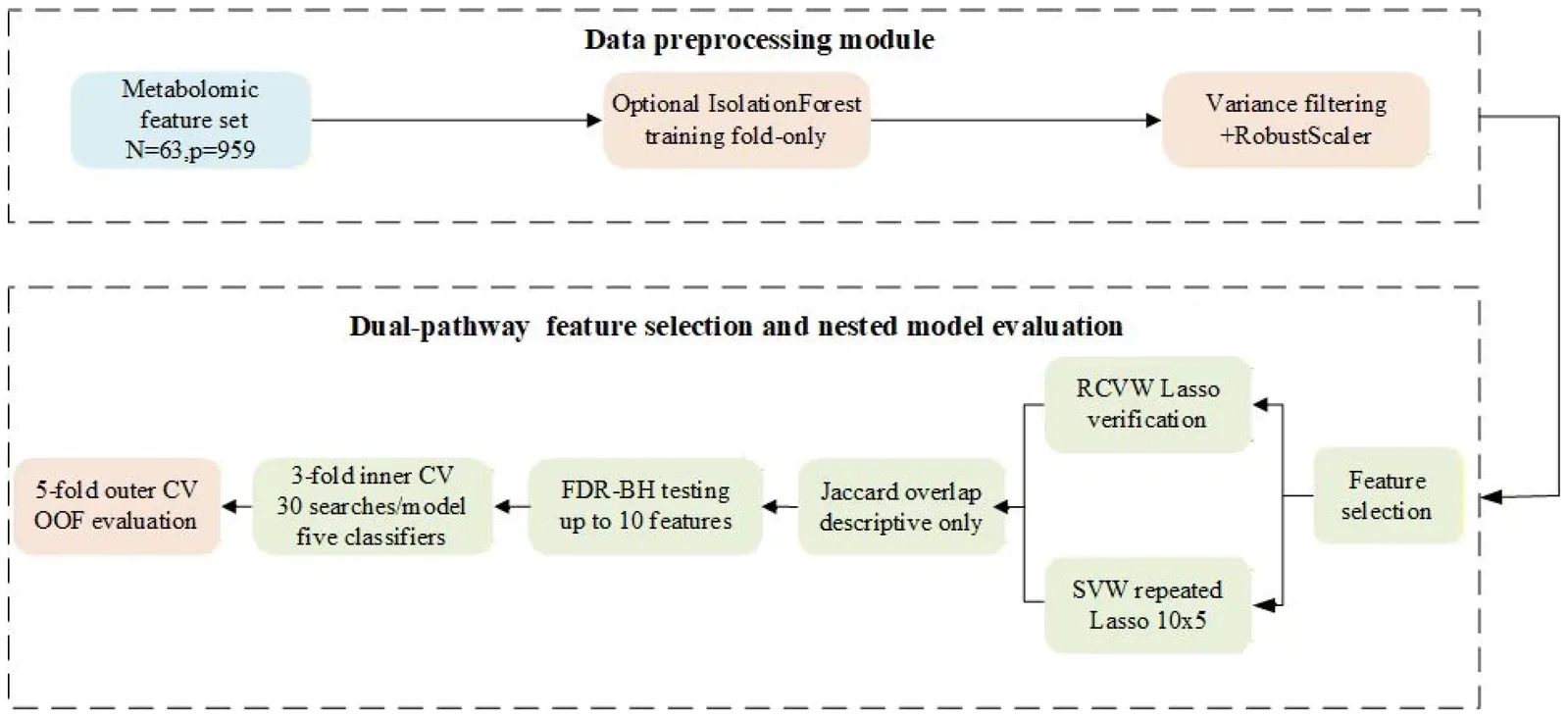
Fold-Contained Nested Cross-Validation Workflow. RCVW: Repeated K-Fold Cross-Validation Way; SVM: Support Vector Machine; CV: Cross-Validation; OOF: Out-of-Fold Evaluation.

The fold-contained nested workflow is illustrated in Figure 2. The fixed model input comprised peak-area measurements for 959 ion features from 63 patients and was constructed before cross-validation as described in Section 2.1.4. Within each outer-training partition generated by this procedure, all subsequent data-dependent model-development procedures, including optional Isolation Forest outlier handling, variance filtering, RobustScaler transformation, repeated cross-validation weighted (RCVW) and single-step validation weighted (SVW) Lasso screening, fold-specific FDR-BH testing, and inner-fold hyperparameter optimization, were fitted using only the corresponding outer-training partition. Thirty sampled hyperparameter configurations were evaluated for each of the five classifiers. The held-out outer-fold samples were used only to generate out-of-fold predictions for performance evaluation.

#### 2.2.1. Data Preprocessing

Data preprocessing comprised optional outlier handling, categorical variable encoding, and robust scaling. Given the small cohort, optional outlier handling was implemented within each relevant training partition using Isolation Forest (n_estimators = 100, max_samples = ‘auto’, random_state = 42) [28]. The primary analysis retained all observations (contamination = 0). To assess sensitivity to outlier removal, the full nested cross-validation workflow was repeated with contamination values of 0.025, 0.05, and 0.10. Categorical clinical covariates were one-hot encoded within each relevant training partition, and the fitted encoder was applied to the corresponding validation or held-out samples.

Continuous features were transformed using RobustScaler, which was fitted within each relevant training partition. The median and interquartile range of the training data were used for scaling, and the fitted transformation was then applied to the corresponding validation or held-out data [29].

#### 2.2.2. Feature Selection Module

To address the high dimensionality and collinearity of untargeted metabolomics data, we designed a dual-pathway feature-selection procedure. The stability-oriented pathway, RCVW, performed repeated Lasso screening via five-fold resampling with 10 repeats within each outer-training partition, using α = 0.02, a maximum of 50,000 iterations, and a tolerance of 0.001, with up to 10 features retained per fit; features selected in at least 25 of the 50 fits were retained. The sparsity-oriented pathway (SVW) applied a single Lasso fit with identical parameters and the same 10-feature cap. Features retained by both pathways were then subjected to fold-specific univariate testing with Benjamini–Hochberg adjustment; if their intersection was empty, the RCVW-derived set was used as the default. Jaccard similarity was calculated to quantify overlap between the two pathways but was not used as a retention threshold. 

#### 2.2.3. Statistical Analysis

Univariate comparisons were performed separately within each outer-training partition after fold-specific feature selection. Normality was assessed within each outcome group using the Shapiro–Wilk test [30]. Welch’s *t* test was used only when both groups met the normality criterion; otherwise, the Mann–Whitney U test was applied [31,32]. All tests were two-sided. Nominal *p* values were adjusted using the Benjamini–Hochberg procedure within each outer-training partition, and q < 0.05 was considered statistically significant. The adjustment family comprised all candidate features tested within the corresponding outer-training partition. *p* values were not pooled across outer folds because each fold represented a separate training-set analysis. Accordingly, the resulting q values are interpreted as fold-specific post-selection evidence rather than as control of a single experiment-wide false-discovery rate across all five folds; recurrence of evidence across folds was summarized separately. The Shapiro–Wilk W statistics, selected test, nominal *p* value, and FDR-adjusted q value are reported for the significant feature-fold comparisons. 

#### 2.2.4. Classifier Selection and Experiment Setup

Five supervised classifiers were compared: Logistic Regression (LR) [33], Support Vector Machine (SVM) [34], Random Forest (RF) [35], Decision Tree (DT) [36], and Extreme Gradient Boosting (XGBoost) [37]. Each model was evaluated using the same outer folds and the corresponding fold-specific feature subset.

A shuffled five-fold outer K-fold procedure was used to estimate internal performance. Hyperparameters were selected by stratified three-fold inner cross-validation. For each model, 30 parameter configurations were sampled and ranked by mean inner-fold AUC. The held-out outer-fold samples were used only for performance estimation.

Performance was summarized across the five outer folds using AUC, accuracy, precision, sensitivity, specificity, and F1 score, and reported as mean ± standard deviation (SD). Pooled out-of-fold probabilities were used to calculate Brier scores and five-bin calibration curves. Sensitivity, specificity, and the Youden index were evaluated at probability thresholds ranging from 0.10 to 0.90, and the prespecified threshold of 0.50 was used to construct confusion matrices. The classifier with the highest mean outer-fold AUC was compared with each remaining classifier using paired non-parametric bootstrap tests with 2000 replicates based on pooled out-of-fold predictions. The resulting four *p* values were adjusted using the Benjamini–Hochberg procedure. The complete parameter search spaces and fold-specific selected hyperparameters are reported in Supplementary Table S1, and the threshold-dependent results are reported in Supplementary Table S2.

### 2.3. Clinical Reference Model and Multi-Modal Comparison

To evaluate the incremental predictive value of metabolomic features beyond routinely available clinical parameters, we prespecified a set of clinical covariates as a baseline reference (see Supplementary Table S4). Covariates were selected according to the following criteria: availability in clinical records, temporal precedence prior to the recurrence event, and plausible relevance to pancreatitis recurrence or metabolic status. Univariate screening or model-derived importance ranking was avoided to prevent data leakage and to reflect real-world clinical practice. The prespecified set comprised demographic variables (age, sex); metabolic variables (BMI, triglycerides, type 2 diabetes, fatty liver); etiological or exposure-related variables (gallstone history, regular alcohol use, smoking history); comorbidity indicators (cardiovascular history); and baseline disease severity (initial acute pancreatitis severity).

To quantify the incremental predictive ability of metabolomic data beyond routine clinical information, we compared metabolomics-only, clinical-only, and combined clinical–metabolomic RF models using the same shuffled five-fold outer cross-validation partitions as in the primary analysis. Missing continuous values were imputed with the training-set median, while missing binary and categorical values were imputed with the training-set mode. Continuous covariates were transformed using RobustScaler, and acute pancreatitis severity was one-hot encoded. All preprocessing parameters were estimated exclusively within the training folds and subsequently applied to the corresponding validation folds. Hyperparameters were selected via stratified three-fold inner cross-validation with 30 randomly sampled configurations. Differences in pooled out-of-fold AUC across the three modality comparisons were evaluated using 2000 paired bootstrap replicates, with Benjamini–Hochberg adjustment applied to the three comparisons. A parallel LR analysis was conducted as a sensitivity analysis to assess the robustness of the results to model choice.

To further characterize the contribution of individual clinical covariates to predictive performance, we performed held-out permutation importance analysis within each outer validation fold. Each covariate was randomly permuted 100 times independently while all other covariates remained unchanged, and importance was quantified as the decrease in validation AUC relative to the unpermuted model. Covariate-level importance was summarized as the mean and standard deviation of the fold-specific mean AUC decreases across the five outer folds.

To explore whether the eight candidate features retained by the dual-pathway selection procedure provided independent information beyond that captured by the prespecified clinical covariates—or merely reflected known clinical factors such as lipid metabolic disturbances or inflammatory severity—we fitted separate Firth-penalized logistic regression models for each candidate metabolite, adjusting for all of the above clinical covariates simultaneously. Continuous variables were standardized, and categorical covariates were entered using dummy (indicator) encoding. Results are reported as adjusted odds ratios and 95% confidence intervals per 1-s.d. increase in candidate feature intensity, with the eight candidate-level *p* values adjusted using the Benjamini–Hochberg procedure. This analysis was positioned as an exploratory post-selection sensitivity analysis and was not treated as independent validation or causal inference.

### 2.4. Cross-Dataset Workflow Portability Analysis

To examine whether the computational workflow could be re-executed on a distinct metabolomics classification task, we analyzed the processed gastric cancer dataset reported by Chen et al. [38]. This dataset was selected because it was publicly available with a prespecified training–test split and a binary metabolomics classification task, allowing the complete workflow to be re-executed without using the RAP labels or tuning on the held-out test set. The gastric cancer cohort was not selected for clinical similarity to the RAP cohort; its different sample size, class balance, feature dimension and disease context were retained as deliberate differences for assessing technical workflow portability. Accordingly, performance in this dataset was interpreted only as evidence of computational re-executability and was not compared directly with the RAP results or used to support RAP biomarker validity. The supplied split comprised 284 training samples, including 187 class-0 and 97 class-1 samples, and 142 held-out test samples, including 94 class-0 and 48 class-1 samples, with 147 metabolomic features. All additional data-dependent processing, dual-pathway Lasso screening, candidate-set FDR adjustment and hyperparameter optimization were restricted to the training partition. For each classifier, 30 parameter configurations were evaluated by stratified three-fold inner cross-validation. The selected models were refitted to the complete training partition, and the held-out test set was evaluated once at a probability threshold of 0.50. AUC confidence intervals and pairwise model comparisons were calculated using 2000 paired bootstrap replicates, with Benjamini–Hochberg adjustment across all ten model comparisons.

## 3. Results

The eight pooled-QC injections showed broadly comparable base-peak chromatographic profiles in both ionization modes, and the QC samples clustered compactly in the PCA score plots, providing qualitative evidence of analytical repeatability (Supplementary Figure S1).

Quantitative QC analysis showed a median feature-level CV of 8.57% (interquartile range, 5.44–14.80%) across the available QC peak-area export (Supplementary Table S7). Overall, 84.9% of the ion features had CV values ≤ 20% and 94.0% had CV values ≤ 30%. The corresponding proportions were 85.9% and 93.0% in positive-ion mode and 83.5% and 95.3% in negative-ion mode, respectively. These measurements provide quantitative evidence of analytical repeatability but do not constitute an explicit feature-level batch-effect or drift-correction procedure.

### 3.1. Fold-Wise Stability of Dual-Pathway Feature Selection

In the primary nested cross-validation analysis, the RCVW and SVW pathways selected 19 and 32 unique features, respectively, across the five outer-training partitions. Nineteen features were shared by the two pathways in at least one partition, and 15 entered a fold-specific final model. The mean fold-wise Jaccard similarity was 0.624, indicating moderate overlap between the two selection pathways. Jaccard similarity was used descriptively and was not applied as a hard threshold for feature retention.

**Figure 3:**
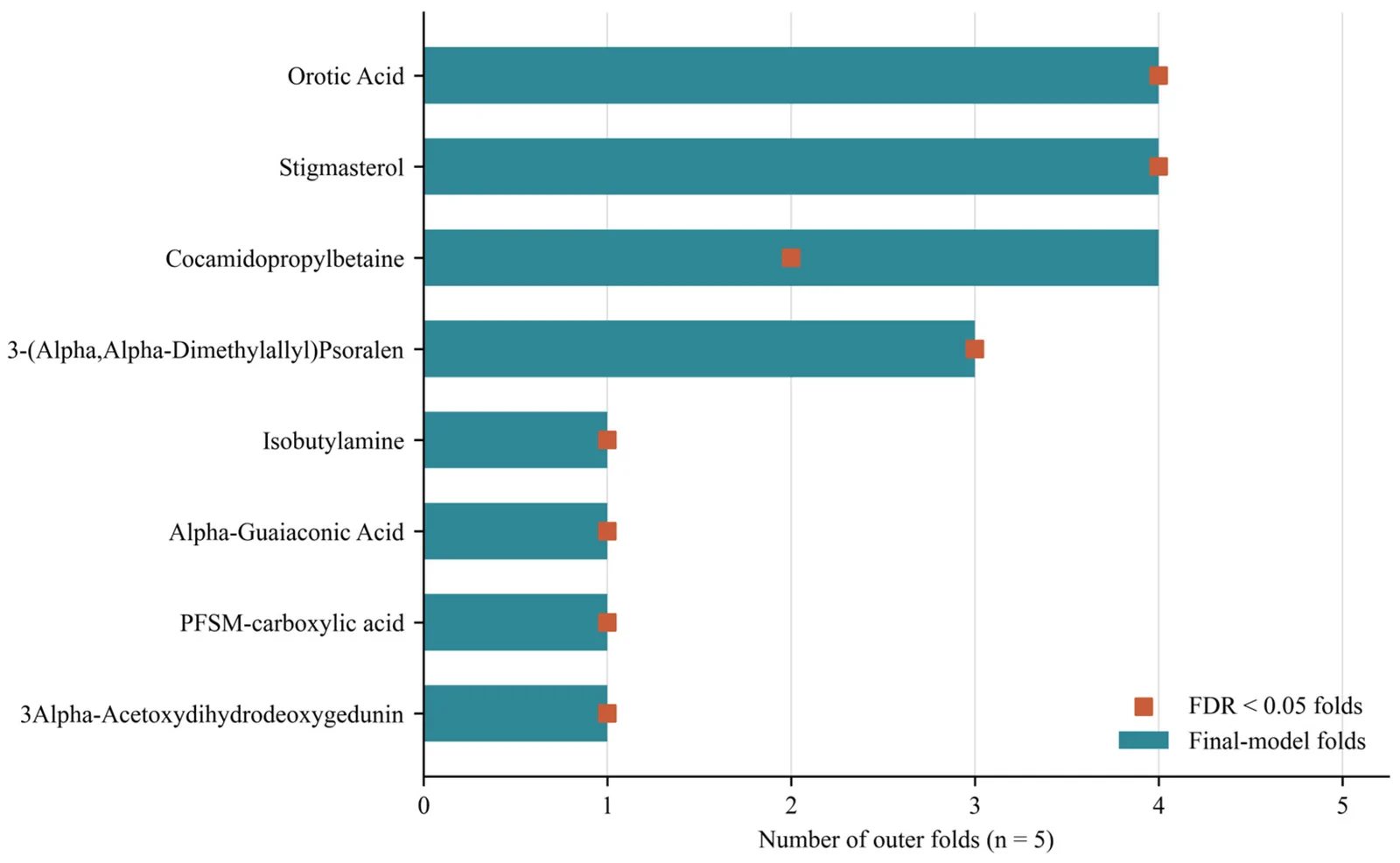
Fold-Wise Stability of the Eight Candidate Features. Bars indicate the number of outer folds in which each feature entered the final model; squares indicate the number of folds in which it met the fold-specific FDR-BH threshold (q < 0.05).

Among the eight candidate annotations that achieved q < 0.05 in at least one outer-training partition, orotic acid, stigmasterol and cocamidopropylbetaine entered the fold-specific final feature set in four of the five partitions, whereas 3-(alpha, alpha-dimethylallyl) psoralen entered in three partitions. Isobutylamine, alpha-guaiaconic acid, PFSM-carboxylic acid and 3alpha-acetoxydihydrodeoxygedunin each entered in only one partition (Figure 3 and Table 2). Orotic acid and stigmasterol met the FDR criterion in all four partitions in which they were selected; 3-(alpha, alpha-dimethylallyl) psoralen met the criterion in all three selected partitions, and cocamidopropylbetaine met the criterion in two of four partitions. Each of the remaining four candidates received FDR support in a single partition. Thus, four of the eight candidates were selected in most outer-training partitions, whereas the other four were supported in only one partition. These results indicate substantial sampling dependence and do not support interpretation of the candidates as a stable biomarker panel. The full-cohort missingness filter excluded only S-D-lactoylglutathione from the earliest 961-feature table, retaining 960 features. When the same rule was fitted independently within the five original outer-training partitions, 960, 960, 959, 959 and 960 features were retained, respectively. The corresponding Jaccard similarities with the full-cohort retained set were 1.000, 1.000, 0.999, 0.999, and 1.000. Pro-Amatine and Amarogentin each exceeded the 50% threshold in one training partition, but neither entered the RCVW, SVW, consensus or final-model feature sets in the primary K-Fold analysis. All eight reported candidate features remained eligible in every outer-training partition. These findings indicate that the practical influence of the upstream filter on feature eligibility was limited (Supplementary Table S3).

### 3.2. Fold-Wise Statistical Evidence for Candidate Features

Fold-specific univariate testing identified 17 comparisons involving eight candidate annotations with q < 0.05 (Table 2 and Table 3). Orotic acid and stigmasterol each met the FDR criterion in four outer-training partitions, 3-(alpha, alpha-dimethylallyl) psoralen met the criterion in three partitions, and cocamidopropylbetaine met the criterion in two partitions. Isobutylamine, alpha-guaiaconic acid, PFSM-carboxylic acid and 3Alpha-Acetoxydihydrodeoxygedunin each met the criterion in one partition. The available annotation metadata and evidence status for these eight candidate features are summarized in Supplementary Table S6. Feature-level mass errors, MS/MS match scores, candidate-specific QC coefficients of variation, blank-sample evidence and authentic-standard confirmation could not be reliably linked to these candidates; accordingly, all eight names are treated as putative annotations rather than confirmed metabolite identities. Normality was assessed separately in the RAP and non-RAP groups using the Shapiro–Wilk test. Because at least one group failed the normality criterion in every listed comparison, all 17 comparisons were evaluated using two-sided Mann–Whitney U tests. Table 3 reports the corresponding Shapiro–Wilk W statistics, nominal *p* values and FDR-adjusted q values. These fold-specific results provide internal evidence for candidate prioritization but do not establish validated biomarkers or causal mechanisms.

No univariate or multivariable logistic regression AUC was estimated from the full 63-patient cohort. Such estimates would reuse the same samples for feature selection and performance assessment and could yield optimistically biased results. Predictive performance was therefore assessed exclusively using the nested cross-validation framework described in Section 3.3.

### 3.3. Nested Cross-Validation Model Comparison 

**Figure 4:**
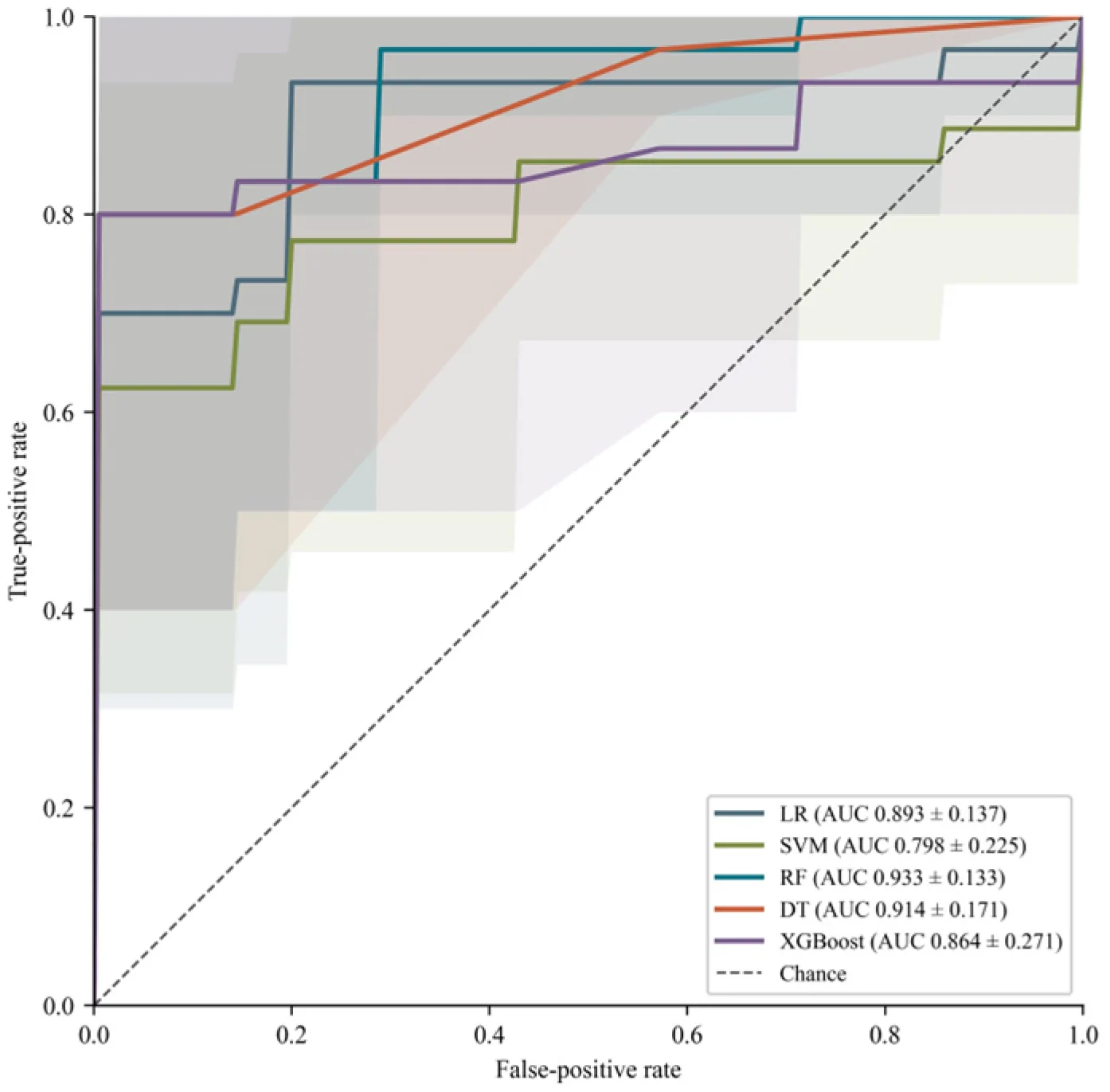
Receiver Operating Characteristic Curves Based on Pooled Out-of-Fold Predicted Probabilities. Legend labels report mean ± SD AUC across the five outer folds. The diagonal dashed line denotes chance discrimination.

**Figure 5:**
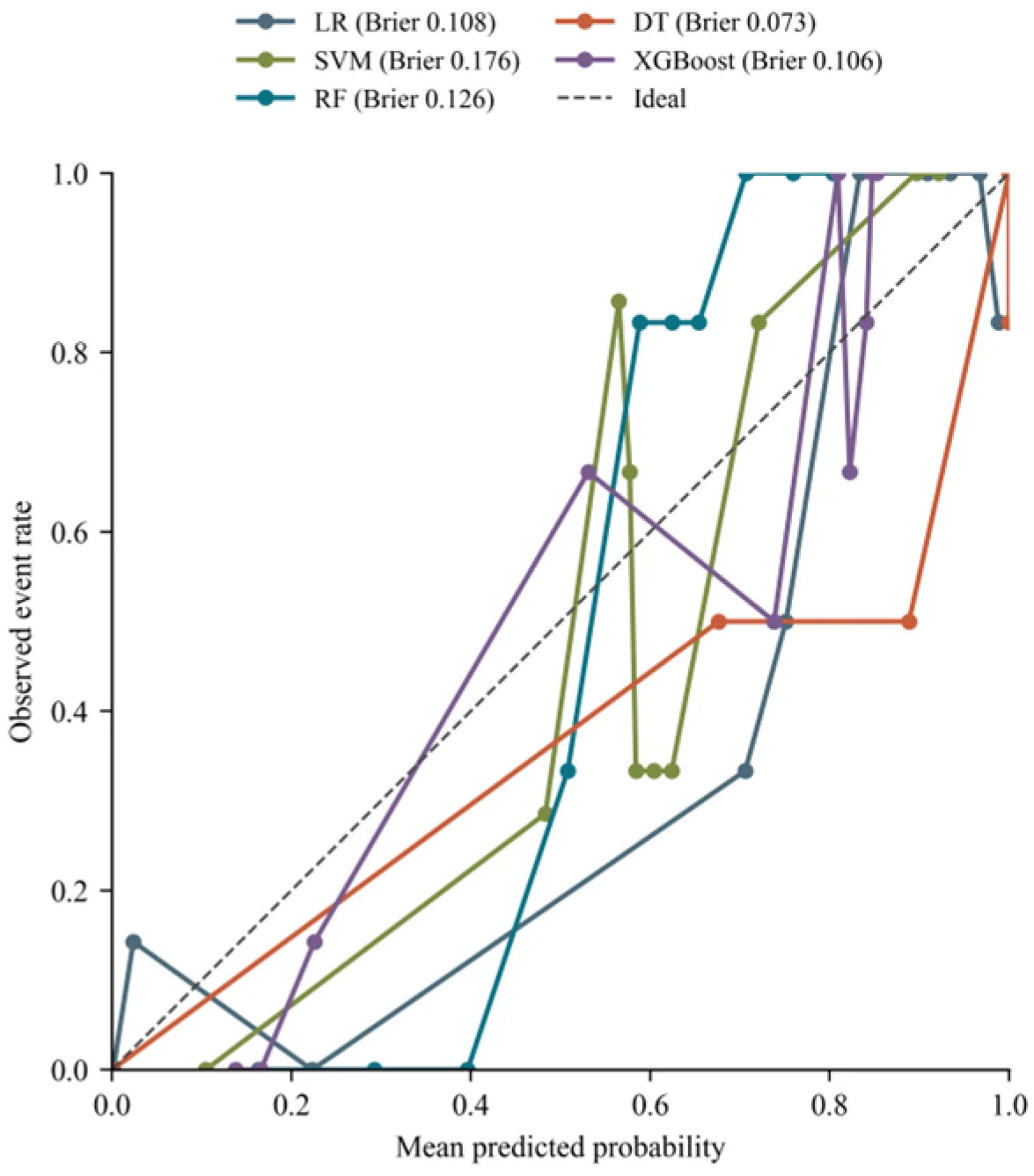
Calibration Curves Based on Pooled Out-of-Fold Predicted Probabilities. The dashed diagonal line denotes ideal calibration, and the legend reports pooled Brier scores.

**Figure 6:**
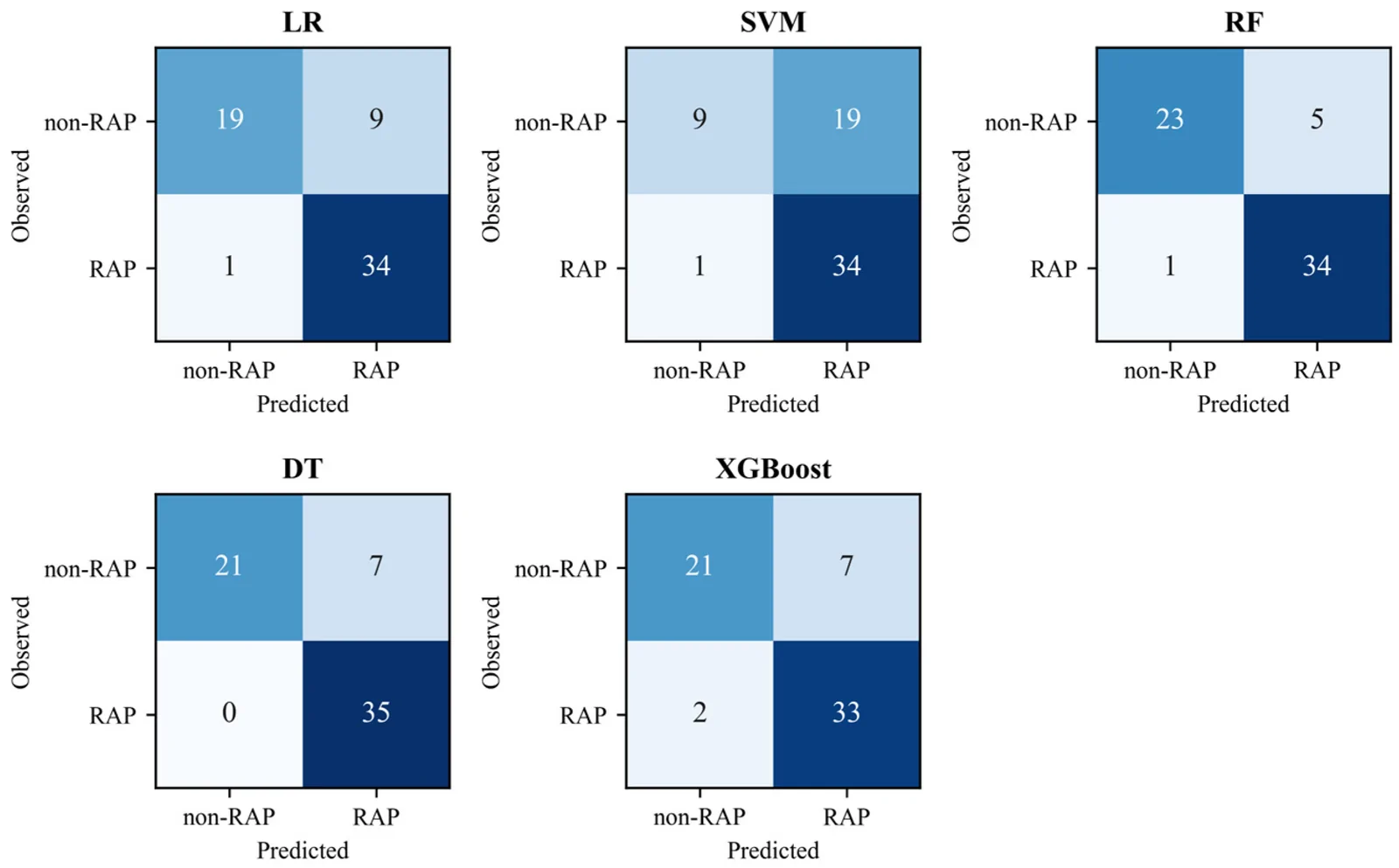
Pooled Out-of-Fold Confusion Matrices for the Five Classifiers. Each matrix comprises predictions for all 63 patients, with each prediction generated when that patient was held out in the corresponding outer fold. Predictions were classified using the prespecified probability threshold of 0.50.

All five classifiers were evaluated under an identical nested cross-validation protocol (Table 4). RF achieved the highest mean outer-fold AUC (0.933 ± 0.133), followed by DT (0.914 ± 0.171) and LR (0.893 ± 0.137). Pairwise bootstrap comparisons based on pooled out-of-fold predictions yielded distinct raw and Benjamini–Hochberg-adjusted *p* values (Supplementary Table S8). RF had a higher pooled out-of-fold AUC than SVM (adjusted *p* = 0.004) but did not differ significantly from XGBoost (adjusted *p* = 0.292), LR (adjusted *p* = 0.773) or DT (adjusted *p* = 0.807). RF therefore had the highest mean outer-fold AUC (0.933 ± 0.133), but its pooled out-of-fold AUC was significantly higher only in comparison with SVM. The ROC curves based on pooled out-of-fold predictions are shown in Figure 4. Calibration analysis of pooled out-of-fold probabilities yielded Brier scores ranging from 0.073 for DT to 0.176 for SVM (Figure 5). At the prespecified probability threshold of 0.50, RF yielded 23 true-negative, 5 false-positive, 1 false-negative and 34 true-positive predictions, whereas SVM yielded 9 true-negative, 19 false-positive, 1 false-negative and 34 true-positive predictions. The corresponding pooled out-of-fold confusion matrices complement the fold-wise mean performance metrics in Table 4 and are shown in Figure 6. Across probability thresholds from 0.10 to 0.90, higher thresholds generally reduced sensitivity while increasing or maintaining specificity, demonstrating the expected classification trade-off (Supplementary Table S2).

Outlier-sensitivity analysis under the same nested cross-validation protocol is summarized in Table 5. At contamination = 0, no samples were removed and RF achieved the highest mean AUC (0.933 ± 0.133). At contamination values of 0.025, 0.05 and 0.10, the highest mean AUCs were achieved by XGBoost (0.815 ± 0.239), SVM (0.752 ± 0.152) and DT (0.778 ± 0.160), respectively. Any level of sample removal therefore reduced the highest observed mean AUC relative to the contamination-free analysis, without showing a monotonic improvement with increasing contamination. The no-removal setting was consequently retained as the primary analysis.

### 3.4. Clinical Covariates and Modality Comparison

Clinical characteristics of the RAP and non-RAP groups are summarized in Supplementary Table S4. Type 2 diabetes was more frequent among patients with RAP than among those without RAP (23/33, 69.7% versus 6/28, 21.4%; Fisher’s exact test, *p* = 0.00026). No other available clinical variable showed evidence of a between-group difference at the nominal 0.05 level.

Under the matched nested cross-validation protocol, the metabolomics-only RF model achieved a mean outer-fold AUC of 0.933 ± 0.133, whereas the clinical-only model achieved an AUC of 0.712 ± 0.265 (Table 6). The combined clinical–metabolomic model achieved a numerically higher AUC of 0.986 ± 0.029. However, the difference in pooled out-of-fold AUC between the combined and metabolomics-only models was not statistically significant (difference, 0.024; 95% confidence interval (CI), −0.003 to 0.065; Benjamini–Hochberg-adjusted *p* = 0.114). Both the combined and metabolomics-only models showed higher pooled out-of-fold AUCs than the clinical-only model (adjusted *p* = 0.0015 for both comparisons).

A parallel LR sensitivity analysis showed the same pattern: the mean AUC was 0.893 ± 0.137 for the metabolomics-only model, 0.590 ± 0.234 for the clinical-only model and 0.915 ± 0.108 for the combined model. The combined versus metabolomics-only comparison was not statistically significant (adjusted *p* = 0.900; Supplementary Table S5). These results indicate that the metabolomic input accounted for most of the observed internal discrimination.

Held-out permutation analysis was used to characterize the predictive contribution of the 11 prespecified clinical covariates (Supplementary Figure S2). Type 2 diabetes showed the largest mean decrease in AUC after permutation (ΔAUC = 0.206 ± 0.174), followed by triglycerides (0.038 ± 0.045) and age (0.030 ± 0.017). The remaining covariates showed small or near-zero mean changes in held-out AUC. These estimates describe predictive contribution within this cohort and should not be interpreted as independent risk effects or causal associations.

In the exploratory post-selection analysis of 57 complete cases, three candidate features retained evidence of association after adjustment for the 11 prespecified clinical covariates and Benjamini–Hochberg correction: orotic acid (adjusted q = 1.04 × 10^−6^), alpha-guaiaconic acid (q = 0.0386) and 3Alpha-acetoxydihydrodeoxygedunin (q = 0.0386; Supplementary Table S9). Because these q values were calculated after supervised Lasso screening, they were interpreted as descriptive post-selection statistics rather than independent evidence of biomarker significance.

### 3.5. Cross-Dataset Robustness Analysis

When the workflow was re-executed on the processed gastric cancer dataset, eight features were retained and all eight met the candidate-set FDR–BH criterion. Because these q values were calculated after the Lasso screening supervised, they are interpreted as descriptive post-selection statistics rather than independent evidence of biomarker significance.

**Figure 7:**
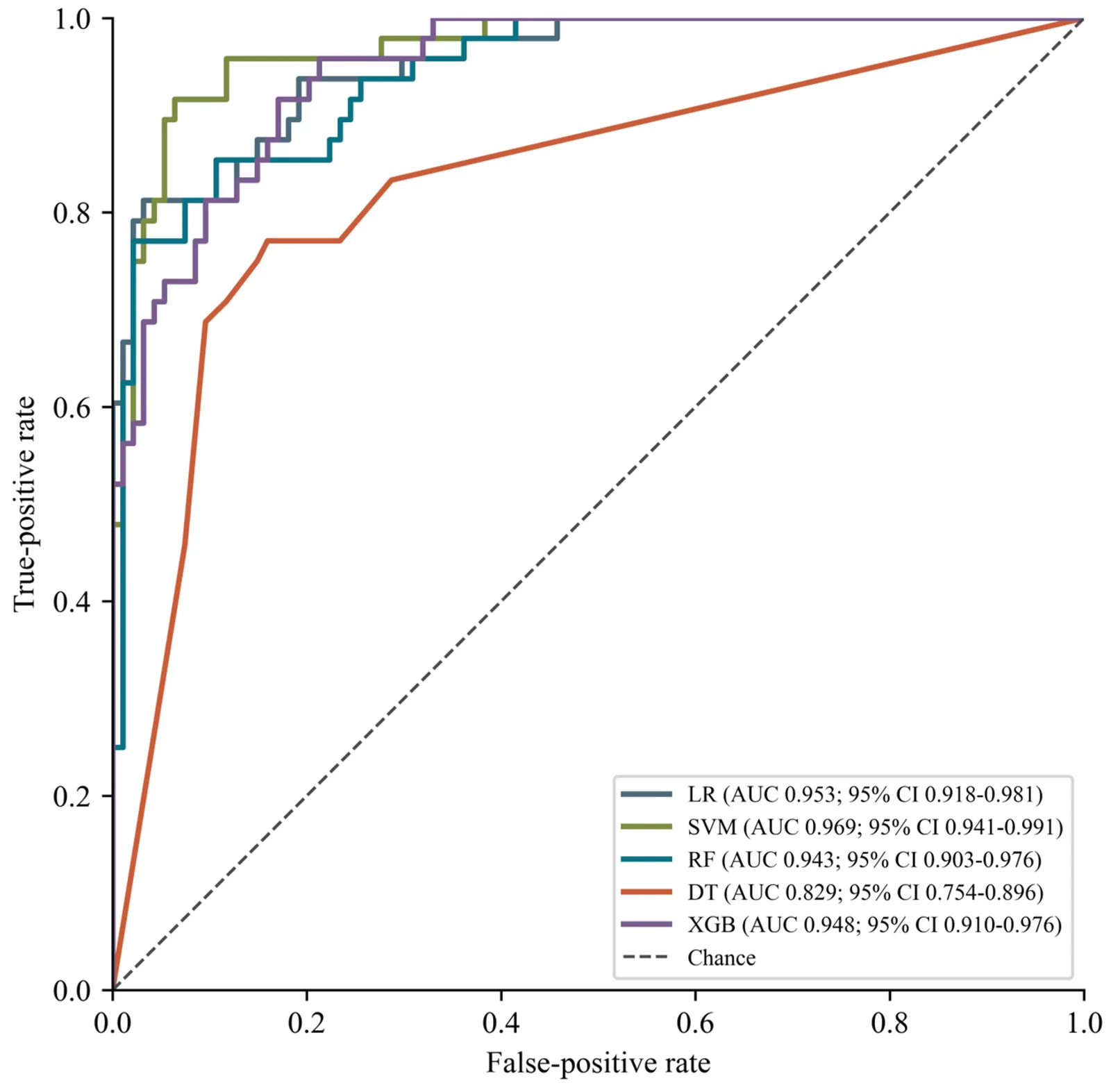
Test-set ROC curves in the cross-dataset workflow portability analysis.

On the prespecified held-out test set, model AUCs ranged from 0.829 to 0.969 (Table 7 and Figure 7). SVM achieved the highest numerical test-set AUC (0.969; 95% CI, 0.941–0.991), with an accuracy of 0.930, sensitivity of 0.917, specificity of 0.936, and Brier score of 0.069. After Benjamini–Hochberg correction across the ten paired bootstrap comparisons, SVM had a higher AUC than RF and DT but did not differ significantly from LR or XGBoost. These results demonstrate that the computational workflow could be re-executed on a distinct processed metabolomics classification task. 

## 4. Discussion

AP remains a major gastrointestinal cause of hospitalization [39,40], and recurrent episodes may progress to chronic pancreatitis and other adverse pancreatic outcomes [41,42,43,44]. Against this clinical need, the principal contribution of IAMFS-RAP is a fold-contained analytical strategy for small, high-dimensional metabolomics studies. After construction of the fixed input matrix, the workflow confines downstream data-dependent model-development procedures, including optional outlier handling, variance filtering, robust scaling, dual-pathway Lasso screening, statistical filtering, and hyperparameter optimization, to the relevant training partitions. This design limits leakage during model development while making the upstream cohort-level feature construction explicit, thereby providing a reproducible basis for evaluating predictive signals without allowing held-out samples to influence downstream model fitting.

The fold-specific missingness audit provided a direct quantitative assessment of the upstream filtering step. Agreement between the full-cohort and outer-training-partition feature sets was nearly complete, with Jaccard similarities ranging from 0.999 to 1.000. Only Pro-Amatine and Amarogentin crossed the missingness threshold in one partition each, and neither entered the primary feature-selection results. All eight reported candidate features remained eligible across every partition. These findings indicate that the practical influence of the upstream filter on feature eligibility and candidate prioritization was limited.

Under this stricter evaluation design, RF achieved the highest mean AUC. Pairwise comparisons based on pooled out-of-fold predictions showed that RF had a higher AUC than SVM after Benjamini–Hochberg correction (adjusted *p* = 0.004), but did not differ significantly from XGBoost, LR, or DT. 

To evaluate the relative contribution of metabolomic data beyond conventional clinical variables, we compared clinical-only, metabolomics-only, and combined RF models. The clinical-only model showed markedly poorer discrimination than its metabolomics-only counterpart. Adding clinical covariates to metabolomics raised the AUC to 0.986 ± 0.029, but this numerical gain was not statistically significant. An LR sensitivity analysis confirmed the same pattern, with no significant improvement over the metabolomics-only model. These results suggest that metabolomic features drove most of the predictive signal in this cohort, whereas the selected clinical variables added little discriminative value. The comparison therefore serves as an exploratory assessment of modality contribution, not as confirmation of clinically meaningful incremental utility.

The observation that several model families retained appreciable discrimination suggests that the internal signal was not dependent on a single algorithm. Moreover, increasingly stringent Isolation Forest removal did not improve performance. This sensitivity analysis provides a practical methodological insight: in small clinical cohorts, unsupervised sample deletion should be evaluated explicitly rather than adopted as a default preprocessing step.

The dual-pathway selection procedure also provided information beyond aggregate model performance. The retained feature panels varied across outer folds, with a mean of 5.4 final-model features per fold, while the two Lasso-based pathways showed moderate agreement (mean Jaccard coefficient = 0.624). Rather than supporting a fixed biomarker panel, these results reveal the sampling sensitivity of feature discovery and provide a transparent basis for prioritizing candidates according to repeated fold-wise support. Orotic acid showed the strongest repeated statistical evidence, making it a candidate for targeted follow-up. More broadly, the framework distinguishes predictive contribution from biochemical validation, thereby supporting candidate prioritization without treating database annotations as confirmed biomarkers or mechanisms.

The annotation audit further defines the boundary of biological interpretation (Supplementary Table S6). Although retention time, *m*/*z*, adduct type, molecular formula, and database identifiers were available, candidate-specific mass errors, MS/MS match scores, QC coefficients of variation, blank sample evidence, and authentic-standard confirmation could not be reliably linked to the eight candidate features. Several candidate labels could plausibly reflect exogenous exposure or analytical contamination rather than endogenous metabolism. Accordingly, these features should be regarded as database-linked putative annotations and prioritized for targeted LC–MS/MS verification using authentic standards, process blanks, and quantitative quality-control measurements before biochemical or clinical interpretation.

Finally, re-execution of the workflow in a public gastric cancer dataset further showed that the computational procedure could be applied to a distinct processed metabolomics classification task. SVM achieved the highest numerical test-set AUC in this dataset, although it did not differ significantly from LR or XGBoost after correction for multiple comparisons. Because feature selection and model fitting were performed anew within the gastric cancer training partition, this analysis supports technical workflow portability.

## 5. Limitations

Several limitations of this study should be acknowledged. First, the single-center small-sample cohort limits statistical precision and generalizability; all results should therefore be interpreted as exploratory internal evidence and require validation in larger independent cohorts. Second, all candidate metabolite annotations are putative, lacking authentic-standard verification and procedural blank confirmation, and some labels may reflect exogenous exposure or analytical contamination rather than endogenous metabolism. Although QC analysis demonstrated acceptable overall repeatability, feature-specific analytical drift or matrix effects cannot be entirely excluded. Third, no independent external RAP cohort was available for predictive validation; the gastric cancer dataset was used solely to assess technical portability of the workflow and does not constitute external validation of RAP prediction. Collectively, this work should be regarded as methodological exploration and candidate-signal prioritization rather than clinical confirmation of biomarkers.

## 6. Conclusions

This study proposes IAMFS-RAP, a fold-contained nested machine learning framework for evaluating recurrence-associated signals in small, high-dimensional metabolomics cohorts. Methodologically, the framework integrates three design features: Isolation Forest-based outlier assessment, a dual-pathway adaptive Lasso strategy that balances stability and sparsity, and strict nested cross-validation to prevent data leakage. In a 63-patient internal cohort, RF achieved the best discriminative performance among five classifiers. The addition of 11 prespecified clinical covariates to the metabolomics-only model yielded a numerically higher AUC (0.986 ± 0.029 vs. 0.933 ± 0.133), with metabolomic features accounting for the predominant share of the predictive signal, though the improvement was not statistically significant. Orotic acid showed the highest selection frequency across outer folds and retained association evidence after covariate adjustment, warranting prioritized targeted validation. Cross-dataset analysis on a public gastric cancer metabolomics cohort further confirmed the technical portability of the workflow to a distinct classification task. Collectively, IAMFS-RAP provides a reproducible methodological basis for metabolomics-based RAP signal prioritization and candidate metabolite selection, though its clinical utility requires independent multicenter cohorts.

## Figures and Tables

**Table 1:** Representative Processed Metabolomic Feature Data.

	Samples	Dibutyl Phthalate	Dimethyl Sulfoxide	…	LPE 18:1	Phenacylamine Hydrochloride	Atalaphylline
Metabolites							
RAP-1		271,891,370.9	48,151,567.15	…	167,320,044.7	149,237,695.8	203,640,436.5
RAP-2		348,560,092.5	47,014,360.47	…	179,428,027.3	139,502,781.9	272,466,486.6
…		…	…	…	…	…	…
RAP-34		327,024,090.6	38,276,741.88	…	45,079,392.32	119,510,724	323,745,099.1
RAP-35		232,039,193.7	30,529,785.37	…	52,926,787.8	176,705,351.4	214,735,804.7
AP-1		5,649,681,745	5,723,254,375	…	84,921,675.43	108,297,279.1	108,682,962.1
AP-2		4,565,963,936	9,444,002,231	…	135,810,274.7	146,821,549.6	209,027,482.6
…		…	…	…	…	…	…
AP-27		7,188,402,117	7,799,584,445	…	130,220,169.9	107,965,225.4	105,909,451.6
AP-28		3,899,948,483	4,317,957,676	…	88,622,584.87	101,432,554.4	227,711,514.3

Note: “AP” denotes patients who did not develop recurrence during the three-month follow-up, whereas “RAP” denotes patients with recurrent acute pancreatitis.

**Table 2:** Fold-Wise Recurrence and FDR Support for Candidate Metabolomic Features.

Feature	Consensus Folds, N	FDR-Positive Folds, N	Median *p*	Median q
Orotic Acid	4	4	1.31 × 10^−9^	8.51 × 10^−9^
Stigmasterol	4	4	3.02 × 10^−3^	7.04 × 10^−3^
Cocamidopropylbetaine	4	2	2.56 × 10^−2^	4.53 × 10^−2^
3-(Alpha, Alpha,Alpha-Dimethylallyl) Psoralen	3	3	1.00 × 10^−2^	2.26 × 10^−2^
Isobutylamine	1	1	1.59 × 10^−4^	5.56 × 10^−4^
Alpha-Guaiaconic Acid	1	1	2.89 × 10^−4^	1.01 × 10^−3^
PFSM-carboxylic acid	1	1	4.76 × 10^−3^	1.43 × 10^−2^
3Alpha-Acetoxydihydrodeoxygedunin	1	1	2.85 × 10^−2^	4.28 × 10^−2^

Note: Consensus folds, number of outer-training partitions in which the feature entered the consensus feature set; FDR-positive folds, number of outer-training partitions in which the feature achieved q < 0.05. Median *p* and q values were calculated across the consensus folds. *p*, nominal two-sided *p* value; q, Benjamini-Hochberg FDR-adjusted *p* value.

**Table 3:** Fold-Wise Normality and FDR-Adjusted Comparisons for Features with q < 0.05.

Feature	Fold	W (Non-RAP)	W (RAP)	Test	*p*	q
Orotic Acid	2	0.630	0.698	Mann–Whitney U	1.31 × 10^−9^	9.16 × 10^−9^
Orotic Acid	3	0.564	0.695	Mann–Whitney U	1.31 × 10^−9^	7.85 × 10^−9^
Orotic Acid	4	0.599	0.657	Mann–Whitney U	1.37 × 10^−9^	1.24 × 10^−8^
Orotic Acid	5	0.626	0.736	Mann–Whitney U	9.03 × 10^−10^	6.32 × 10^−9^
Stigmasterol	2	0.557	0.683	Mann–Whitney U	4.71 × 10^−3^	1.10 × 10^−2^
Stigmasterol	3	0.495	0.819	Mann–Whitney U	2.47 × 10^−2^	4.94 × 10^−2^
Stigmasterol	4	0.588	0.814	Mann–Whitney U	1.69 × 10^−4^	7.59 × 10^−4^
Stigmasterol	5	0.548	0.736	Mann–Whitney U	1.32 × 10^−3^	3.08 × 10^−3^
Cocamidopropylbetaine	4	0.609	0.908	Mann–Whitney U	2.22 × 10^−2^	3.99 × 10^−2^
Cocamidopropylbetaine	5	0.677	0.917	Mann–Whitney U	1.75 × 10^−2^	3.07 × 10^−2^
3-(Alpha,Alpha-Dimethylallyl) Psoralen	1	0.520	0.314	Mann–Whitney U	3.19 × 10^−3^	9.58 × 10^−3^
3-(Alpha,Alpha-Dimethylallyl) Psoralen	3	0.508	0.319	Mann–Whitney U	1.34 × 10^−2^	4.03 × 10^−2^
3-(Alpha,Alpha-Dimethylallyl) Psoralen	4	0.498	0.230	Mann–Whitney U	1.00 × 10^−2^	2.26 × 10^−2^
Isobutylamine	2	0.553	0.828	Mann–Whitney U	1.59 × 10^−4^	5.56 × 10^−4^
Alpha-Guaiaconic Acid	5	0.836	0.638	Mann–Whitney U	2.89 × 10^−4^	1.01 × 10^−3^
PFSM-carboxylic acid	4	0.846	0.879	Mann–Whitney U	4.76 × 10^−3^	1.43 × 10^−2^
3Alpha-Acetoxydihydrodeoxygedunin	4	0.804	0.784	Mann–Whitney U	2.85 × 10^−2^	4.28 × 10^−2^

**W**, Shapiro–Wilk statistic; ***p***, unadjusted *p* value; **q**, Benjamini–Hochberg-adjusted *p* value. FDR correction was applied independently within each outer-training fold. Only comparisons with q < 0.05 are shown.

**Table 4:** Nested Cross-Validation Performance of the five Classifiers.

Model	AUC	Accuracy	Precision	Sensitivity	Specificity	F1-Score
LR	0.893 ± 0.137	0.845 ± 0.233	0.836 ± 0.216	0.967 ± 0.067	0.720 ± 0.371	0.886 ± 0.168
SVM	0.798 ± 0.225	0.681 ± 0.221	0.680 ± 0.224	0.971 ± 0.057	0.400 ± 0.420	0.775 ± 0.151
RF	0.933 ± 0.133	0.908 ± 0.185	0.900 ± 0.200	0.967 ± 0.067	0.857 ± 0.286	0.925 ± 0.150
DT	0.914 ± 0.171	0.892 ± 0.215	0.892 ± 0.215	1.000 ± 0.000	0.800 ± 0.400	0.926 ± 0.147
XGBoost	0.864 ± 0.271	0.862 ± 0.240	0.858 ± 0.233	0.933 ± 0.133	0.789 ± 0.332	0.888 ± 0.195

**Table 5:** Outlier-Sensitivity Analysis under the Same Nested Cross-Validation Workflow.

Contamination	Best Model	AUC	Accuracy	Sensitivity	Specificity
0.000	RF	0.933 ± 0.133	0.908 ± 0.185	0.967 ± 0.067	0.857 ± 0.286
0.025	XGBoost	0.815 ± 0.239	0.810 ± 0.213	0.876 ± 0.152	0.737 ± 0.270
0.05	SVM	0.752 ± 0.152	0.672 ± 0.197	0.881 ± 0.168	0.360 ± 0.445
0.10	Decision Tree	0.778 ± 0.160	0.686 ± 0.165	0.667 ± 0.222	0.766 ± 0.225

Values are mean ± SD across the five outer folds. The best model at each contamination setting was selected according to mean outer-fold AUC. At contamination = 0, no samples were removed.

**Table 6:** Nested Cross-Validation Performance of Metabolomics-Only, Clinical-Only and Combined Random Forest Models.

Model	AUC	Accuracy	Precision	Sensitivity	Specificity	F1
Metabolomics-only	0.933 ± 0.133	0.908 ± 0.185	0.900 ± 0.200	0.967 ± 0.067	0.857 ± 0.286	0.925 ± 0.150
Clinical-only	0.712 ± 0.265	0.619 ± 0.219	0.627 ± 0.214	0.763 ± 0.243	0.434 ± 0.329	0.677 ± 0.208
Combined	0.986 ± 0.029	0.877 ± 0.210	0.870 ± 0.209	1.000 ± 0.000	0.760 ± 0.388	0.915 ± 0.143

Values are mean ± SD across the five outer folds. The prespecified probability threshold of 0.50 was used for threshold-dependent metrics. All three models used identical shuffled outer folds, training-contained preprocessing and stratified three-fold inner hyperparameter optimization.

**Table 7:** Test-Set Performance in the Cross-Dataset Workflow Portability Analysis.

Model	AUC (95% CI)	Acc.	Prec.	Sens.	Spec.	F1	Brier	Adjusted *p* vs. SVM
LR	0.953 (0.918–0.981)	0.866	0.796	0.813	0.894	0.804	0.083	0.293
SVM	0.969 (0.941–0.991)	0.930	0.880	0.917	0.936	0.898	0.069	-
RF	0.943 (0.903–0.976)	0.873	0.813	0.813	0.904	0.813	0.092	0.012
DT	0.829 (0.754–0.896)	0.803	0.685	0.771	0.819	0.725	0.170	0.0025
XGBoost	0.948 (0.910–0.976)	0.852	0.765	0.813	0.872	0.788	0.093	0.160

Note: Performance values are point estimates from the fixed test set (*n* = 142) at a probability threshold of 0.50 and are therefore not reported as mean ± SD. AUC 95% CIs were estimated with 2000 non-parametric bootstrap samples. Adjusted *p* values were derived from paired bootstrap tests comparing each model to SVM and adjusted across all ten pairwise comparisons using the Benjamini–Hochberg procedure. Class 1 was treated as the positive class.

## Data Availability

Restricted by privacy and ethical regulations, the data of this study cannot be made publicly available. The relevant codes and raw data can be obtained from the corresponding author upon email request.

## Supplementary Materials

The following supporting information can be downloaded at: https://www.mdpi.com/article/10.3390/metabo16080575/s1, Supplementary Table S1, Hyperparameter search spaces and fold-specific selected configurations for the primary nested cross-validation analysis (contamination = 0); Supplementary Table S2, Threshold-dependent sensitivity, specificity and Youden index based on pooled out-of-fold predictions; Supplementary Table S3, Fold-specific audit of the upstream outcome-group-informed missingness filter; Supplementary Table S4, Baseline clinical characteristics of the RAP and non-RAP groups; Supplementary Table S5, Logistic-regression sensitivity analysis of metabolomics-only, clinical-only and combined models; Supplementary Table S6, Annotation evidence for eight candidate features; Supplementary Table S7, Quantitative QC repeatability metrics; Supplementary Table S8, Pairwise AUC comparisons with Random Forest; Supplementary Table S9, Exploratory clinical covariate-adjusted associations for the eight candidate features; Supplementary Figure S1, Analytical quality-control assessment of the untargeted LC-MS/MS run. (a,b) Base-peak chromatograms obtained from eight pooled-QC injections in positive- and negative-ion modes, respectively. (c,d) PCA score plots of the complete analytical dataset in positive- and negative-ion modes, respectively. The broadly comparable chromatographic profiles and compact clustering of QC samples provide qualitative evidence of analytical repeatability; Supplementary Figure S2, Held-out permutation importance of prespecified clinical covariates. Permutation importance was evaluated using held-out data within each of the five outer cross-validation folds. Bars show the mean decrease in AUC after permuting each prespecified clinical covariate, and error bars denote the standard deviation across outer folds. The clinical-only random-forest model achieved a mean outer-fold AUC of 0.712 ± 0.265. Larger positive ΔAUC values indicate a greater contribution to held-out discrimination. Values close to zero or below zero indicate that permuting the covariate did not consistently reduce held-out AUC in this small cohort and should not be interpreted as evidence for a protective clinical association.

## Abbreviations

The following abbreviations are used in this manuscript:

BMI	Body Mass Index
ALFS	Adaptive Lasso-based Feature Selection Strategy
AP	Acute Pancreatitis
AUC	Area Under the Receiver Operating Characteristic Curve
CP	Chronic Pancreatitis
DT	Decision Tree
CI	Confidence Interval
HESI	Heated Electrospray Ionization
HMDB	Human Metabolome Database
IAMFS-RAP	Integrated Adaptive Metabolomic Feature Selection and Prediction for Recurrent Acute Pancreatitis
CV	Coefficient of Variation
LC–MS/MS	Liquid Chromatography–Tandem Mass Spectrometry
LR	Logistic Regression
FDR	False Discovery Rate
OR	Odds Ratio
PCA	Principal Component Analysis
PDAC	Pancreatic Ductal Adenocarcinoma
PLS-DA	Partial Least Squares Discriminant Analysis
QC	Quality Control
RAP	Recurrent Acute Pancreatitis
RCVW	Repeated Cross-Validation Weighted
RF	Random Forest
ROC	Receiver Operating Characteristic
OOF	Out of Fold
SVM	Support Vector Machine
SVW	Single-step Validation Weighted
UHPLC	Ultra-High-Performance Liquid Chromatography
UPLC-MS/MS	Ultra-High-Performance Liquid Chromatography–Tandem Mass Spectrometry
SD	Standard Deviation
XGBoost	Extreme Gradient Boosting
