# Supplementary material for: A Metabolomics-Driven Integrated Machine Learning Framework for Early Prediction of Recurrent Acute Pancreatitis
Source: Metabolites. 2026 Aug 15;16(8):575. doi: 10.3390/metabo16080575 (PMC13515787; doi:10.3390/metabo16080575)
Supplement: Supplementary file 1 [file metabolites-16-00575-s001.zip › metabolites-4438105-supplementary.pdf]

Supplementary Table S1. Hyperparameter search spaces and fold-specific selected configurations for the primary nested cross-validation analysis (contamination = 0).

For each model and outer fold, 30 parameter configurations were sampled using ParameterSampler and evaluated by stratified 3-fold inner cross-validation. The configuration with the highest mean inner-fold ROC-AUC was selected. Continuous ranges indicate uniform sampling; integer ranges are inclusive.

Panel A. Hyperparameter search spaces

Model	Hyperparameter search space
Logistic Regression	C, 0.01-10.01; penalty, l1 or l2; solver, liblinear; max_iter, 2,000, 3,000 or 5,000
Supported vector machine	C, 0.01-5.01; kernel, linear, rbf or poly; gamma, scale, auto, or one of 0.0790, 0.1883, 0.3003, 0.3670 and 0.4764; class_weight, balanced or None
Random Forest	n_estimators, 100-500; max_depth, 1, 2, 3, 5, 7, 10 or None; min_samples_split, 2-19; min_samples_leaf, 1-14; max_features, 0.1, 0.3, 0.5, sqrt, log2 or None; class_weight, balanced, balanced_subsample or None; bootstrap, True or False
Decision Tree	max_depth, 2, 3, 5, 7 or 10; min_samples_split, 5-14; min_samples_leaf, 3-9; max_features, sqrt, log2 or None; class_weight, balanced or None; ccp_alpha, 0.005-0.030
Extreme Gradient Boosting	n_estimators, 50-300; max_depth, 1-7; learning_rate, 0.01-0.31; subsample, 0.60-1.00; colsample_bytree, 0.60-1.00; reg_alpha, 0-3; reg_lambda, 0-5; min_child_weight, 1-5; gamma, 0-1.5

Panel B. Fold-specific selected configurations

Model	Outer fold	Selected configuration
Logistic regression	1	C=1.5702; penalty=l1; solver=liblinear; max_iter=5,000
Logistic regression	2	C=0.3539; penalty=l1; solver=liblinear; max_iter=3,000
Logistic regression	3	C=0.5908; penalty=l2; solver=liblinear; max_iter=2,000
Logistic regression	4	C=1.5702; penalty=l1; solver=liblinear; max_iter=5,000
Logistic regression	5	C=1.4387; penalty=l2; solver=liblinear; max_iter=5,000
Support vector machine	1	C=1.0717; kernel=linear; gamma=0.4764; class_weight=None
Support vector machine	2	C=0.7900; kernel=linear; gamma=0.1883; class_weight=balanced
Support vector machine	3	C=1.4147; kernel=linear; gamma=0.3670; class_weight=None
Support vector machine	4	C=4.3097; kernel=linear; gamma=0.3670; class_weight=balanced
Support vector machine	5	C=3.0974; kernel=linear; gamma=0.3003; class_weight=None
Random forest	1	n_estimators=108; max_depth=7; min_samples_split=8; min_samples_leaf=9; max_features=0.1; class_weight=None; bootstrap=True
Random forest	2	n_estimators=154; max_depth=None; min_samples_split=13; min_samples_leaf=3; max_features=None; class_weight=balanced_subsample; bootstrap=False

Random forest	3	n_estimators=269; max_depth=3; min_samples_split=3; min_samples_leaf=6; max_features=0.5; class_weight=None; bootstrap=False
Random forest	4	n_estimators=140; max_depth=1; min_samples_split=14; min_samples_leaf=12; max_features=sqrt; class_weight=balanced_subsample; bootstrap=True
Random forest	5	n_estimators=330; max_depth=1; min_samples_split=11; min_samples_leaf=5; max_features=0.5; class_weight=None; bootstrap=True
Decision tree	1	max_depth=10; min_samples_split=14; min_samples_leaf=4; max_features=None; class_weight=None; ccp_alpha=0.0187
Decision tree	2	max_depth=5; min_samples_split=12; min_samples_leaf=3; max_features=None; class_weight=None; ccp_alpha=0.0243
Decision tree	3	max_depth=5; min_samples_split=8; min_samples_leaf=7; max_features=None; class_weight=balanced; ccp_alpha=0.0161
Decision tree	4	max_depth=5; min_samples_split=8; min_samples_leaf=3; max_features=log2; class_weight=None; ccp_alpha=0.0202
Decision tree	5	max_depth=5; min_samples_split=12; min_samples_leaf=3; max_features=None; class_weight=None; ccp_alpha=0.0243

Extreme gradient boosting	1	n_estimators=137; max_depth=5; learning_rate=0.2053; subsample=0.6727; colsample_bytree=0.7335; reg_alpha=2.4973; reg_lambda=1.0617; min_child_weight=2; gamma=0.2143
Extreme gradient boosting	2	n_estimators=197; max_depth=7; learning_rate=0.2233; subsample=0.8764; colsample_bytree=0.7304; reg_alpha=1.9355; reg_lambda=0.8718; min_child_weight=3; gamma=0.3304
Extreme gradient boosting	3	n_estimators=177; max_depth=3; learning_rate=0.2333; subsample=0.7471; colsample_bytree=0.6715; reg_alpha=1.7049; reg_lambda=0.4684; min_child_weight=1; gamma=0.5497
Extreme gradient boosting	4	n_estimators=194; max_depth=3; learning_rate=0.2116; subsample=0.8529; colsample_bytree=0.9943; reg_alpha=2.1846; reg_lambda=1.8389; min_child_weight=3; gamma=0.3631
Extreme gradient boosting	5	n_estimators=109; max_depth=4; learning_rate=0.0295; subsample=0.6064; colsample_bytree=0.8430; reg_alpha=1.6899; reg_lambda=1.9271; min_child_weight=1; gamma=0.2558

C, inverse regularization strength; max_iter, maximum number of iterations; ccp_alpha, complexity parameter for minimal cost-complexity pruning; n_estimators, number of trees; reg_alpha and reg_lambda, L1 and L2 regularization terms, respectively. Gamma is reported for the sampled SVM configurations although it is not operative when the linear kernel is selected.

Supplementary Table S2. Threshold-dependent sensitivity, specificity and Youden index based on pooled out-of-fold predictions.

Model	Threshold	Sensitivity	Specificity	Youden index
LR	0.10	0.971	0.393	0.364
LR	0.20	0.943	0.607	0.550
LR	0.30	0.914	0.679	0.593
LR	0.40	0.914	0.786	0.700
LR	0.50	0.914	0.821	0.736
LR	0.60	0.886	0.857	0.743
LR	0.70	0.829	0.929	0.757
LR	0.80	0.600	0.929	0.529
LR	0.90	0.314	1.000	0.314
SVM	0.10	1.000	0.393	0.393
SVM	0.20	1.000	0.536	0.536
SVM	0.30	1.000	0.571	0.571
SVM	0.40	1.000	0.643	0.643

**SVM**	**0.50**	**0.971**	**0.643**	**0.614**
**SVM**	**0.60**	**0.800**	**0.857**	**0.657**
**SVM**	**0.70**	**0.800**	**0.893**	**0.693**
**SVM**	**0.80**	**0.743**	**1.000**	**0.743**
**SVM**	**0.90**	**0.571**	**1.000**	**0.571**
**RF**	**0.10**	**1.000**	**0.214**	**0.214**
**RF**	**0.20**	**1.000**	**0.357**	**0.357**
**RF**	**0.30**	**1.000**	**0.429**	**0.429**
**RF**	**0.40**	**0.943**	**0.679**	**0.621**
**RF**	**0.50**	**0.914**	**0.821**	**0.736**
**RF**	**0.60**	**0.886**	**0.964**	**0.850**
**RF**	**0.70**	**0.686**	**0.964**	**0.650**
**RF**	**0.80**	**0.514**	**0.964**	**0.479**
**RF**	**0.90**	**0.286**	**1.000**	**0.286**
**DT**	**0.10**	**0.943**	**0.679**	**0.621**

**DT**	**0.20**	**0.943**	**0.679**	**0.621**
**DT**	**0.30**	**0.914**	**0.750**	**0.664**
**DT**	**0.40**	**0.886**	**0.821**	**0.707**
**DT**	**0.50**	**0.886**	**0.821**	**0.707**
**DT**	**0.60**	**0.886**	**0.821**	**0.707**
**DT**	**0.70**	**0.857**	**0.964**	**0.821**
**DT**	**0.80**	**0.857**	**0.964**	**0.821**
**DT**	**0.90**	**0.857**	**0.964**	**0.821**
**XGB**	**0.10**	**1.000**	**0.000**	**0.000**
**XGB**	**0.20**	**1.000**	**0.357**	**0.357**
**XGB**	**0.30**	**0.971**	**0.679**	**0.650**
**XGB**	**0.40**	**0.971**	**0.750**	**0.721**
**XGB**	**0.50**	**0.914**	**0.750**	**0.664**
**XGB**	**0.60**	**0.829**	**0.893**	**0.721**
**XGB**	**0.70**	**0.743**	**1.000**	**0.743**

**XGB**	**0.80**	**0.486**	**1.000**	**0.486**
**XGB**	**0.90**	**0.000**	**1.000**	**0.000**

Values were calculated from the pooled out-of-fold predictions generated by the primary nested cross-validation analysis with contamination = 0. RAP was treated as the positive class. Thresholds were applied after model evaluation and did not involve model refitting. The Youden index was calculated as sensitivity + specificity − 1. Values are pooled point estimates for all 63 patients and are therefore not presented as mean ± SD. LR, logistic regression; SVM, support vector machine; RF, Random Forest; DT, Decision Tree; XGB, Extreme Gradient Boosting.

Supplementary Table S3. Fold-specific audit of the upstream outcome-group-informed missingness filter.

Outer fold	Training n	non-RAP/RAP	Retained features	Additional removal	Jaccard	Candidates retained
**1**	50	21/29	960	None	1.000	8/8
**2**	50	24/26	960	None	1.000	8/8
**3**	50	23/27	959	Pro-Amatine	0.999	8/8
**4**	51	21/30	959	Amarogentin	0.999	8/8
**5**	51	23/28	960	None	1.000	8/8

Feature counts refer to the earliest available 961-feature processed table. The full-cohort missingness filter excluded S-D-lactoylglutathione. One additional feature, Phthalic Anhydride, was not carried into the fixed 959-feature model matrix independently of the missingness rule. Jaccard similarity compares the fold-specific and full-cohort retained feature sets. The eight candidates were Orotic Acid, Stigmasterol, Cocamidopropylbetaine, 3-(Alpha, Alpha-Dimethylallyl) Psoralen, Isobutylamine, Alpha-Guaiaconic Acid, PFSM-carboxylic acid and 3Alpha-Acetoxydihydrodeoxygedunin.

Supplementary Table S4. Baseline clinical characteristics of the RAP and non-RAP groups.

Characteristic	Non-RAP (n = 28)	RAP (n = 35)	P value	Test
**Age, years**	35.00 (32.75–40.00)	33.00 (28.50–37.00)	0.114	Mann–Whitney U
**BMI, kg m^{-2}**	27.61 (25.81–30.66)	29.00 (26.82–31.52)	0.205	Mann–Whitney U
**Triglycerides, mmol L^{-1}**	10.02 (6.35–17.76)	15.33 (11.43–20.24)	0.057	Mann–Whitney U
**Male sex**	24/28 (85.7%)	29/35 (82.9%)	1.000	Fisher's exact
**Type 2 diabetes**	6/28 (21.4%)	23/33 (69.7%)	0.00026	Fisher's exact
**History of gallstones**	1/28 (3.6%)	0/35 (0.0%)	0.444	Fisher's exact
**Cardiovascular history**	7/28 (25.0%)	7/35 (20.0%)	0.763	Fisher's exact
**Smoking history**	15/28 (53.6%)	15/35 (42.9%)	0.453	Fisher's exact
**Regular alcohol use**	6/28 (21.4%)	6/35 (17.1%)	0.752	Fisher's exact
**Fatty liver**	24/28 (85.7%)	30/35 (85.7%)	1.000	Fisher's exact
**AP severity: mild**	20/28 (71.4%)	24/35 (68.6%)	0.086	Chi-square
**AP severity: moderate**	5/28 (17.9%)	11/35 (31.4%)	—	—
**AP severity: severe**	3/28 (10.7%)	0/35 (0.0%)	—	—

Values are presented as median (interquartile range) for continuous variables and n/N (%) for categorical variables. Denominators reflect available observations; BMI was available for 58 patients and type 2 diabetes status for 61 patients, whereas triglyceride measurements were available for all 63 patients. P values are two-sided. Continuous variables were compared using the Mann–Whitney U test. For acute pancreatitis severity, the reported P value and test refer to the overall comparison across the three severity categories. BMI, body mass index; AP, acute pancreatitis; RAP, recurrent acute pancreatitis.

Supplementary Table S5. Logistic-regression sensitivity analysis of metabolomics-only, clinical-only and combined models.

Model	AUC	Accuracy	Precision	Sensitivity	Specificity	F1	Brier score
Metabolomics-only	0.893 ± 0.137	0.845 ± 0.233	0.836 ± 0.216	0.967 ± 0.067	0.720 ± 0.371	0.886 ± 0.168	0.107 ± 0.137
Clinical-only	0.590 ± 0.234	0.582 ± 0.175	0.599 ± 0.175	0.672 ± 0.215	0.471 ± 0.116	0.627 ± 0.182	0.261 ± 0.087
Combined	0.915 ± 0.108	0.873 ± 0.128	0.853 ± 0.146	0.887 ± 0.157	0.846 ± 0.158	0.864 ± 0.144	0.101 ± 0.101

Values are mean ± SD across the five outer folds. The analysis included the 11 prespecified baseline clinical covariates: age, sex, BMI, triglycerides, type 2 diabetes, gallstone history, cardiovascular history, smoking history, regular alcohol use, fatty liver and initial acute pancreatitis severity. The prespecified probability threshold of 0.50 was used for threshold-dependent metrics. All preprocessing transformations and hyperparameter selection were performed within the corresponding training partitions. The pooled out-of-fold AUC difference between the combined and metabolomics-only models was 0.008 (95% CI, −0.071 to 0.096; Benjamini–Hochberg-adjusted P = 0.900). Brier scores were calculated from pooled out-of-fold probabilities. AUC, area under the receiver operating characteristic curve; SD, standard deviation; BMI, body mass index.

Supplementary Table S6. Annotation evidence for eight candidate features.

Candidate	ID	RT (min)	m/z	Adduct	Formula	HMDB	KEGG	Mode	Error (ppm)	MS/MS score	QC CV (%)	Blank	Std.	Status
Orotic Acid	P3039	1.933	157.03435	$[M+H]^+$	C5H4N2O4	HMDB0000226	C00295	Positive	NA	NA	NA	NA	No	Putative annotation
Stigmasterol	P11628	11.970	435.36475	$[M+H]^+$	C29H48O	HMDB0000937	C05442	Positive	NA	NA	NA	NA	No	Putative annotation
Cocamidopropylbetaine	P9707	7.724	343.29413	$[M+H]^+$	C19H38N2O3	Not assigned	Not assigned	Positive	NA	NA	NA	NA	No	Putative annotation
3-(Alpha,Alpha-Dimethylallyl) Psoralen	P7496	5.224	272.13406	$[M+H-H_2O]^+$	C16H14O3	Not assigned	C09165	Positive	NA	NA	NA	NA	No	Putative annotation
Isobutylamine	P105	12.838	74.09675	$[M+H]^+$	C4H11N	HMDB0034198	C02787	Positive	NA	NA	NA	NA	No	Putative annotation

Alpha-Guaiaconic Acid	N7610	6.989	339.13370	$[M-H]^-$	C20H20O5	Not assigned	Not assigned	Negative	NA	NA	NA	NA	No	Putative annotation
PFSM-carboxylic acid	N7026	6.107	319.98227	$[M-H]^-$	C6H6F7NO4S	Not assigned	Not assigned	Negative	NA	NA	NA	NA	No	Putative annotation
3Alpha-Acetoxydihydrodeoxygedunin	P14222	13.142	535.37433	$[M+H]^+$	C30H40O7	Not assigned	Not assigned	Positive	NA	NA	NA	NA	No	Putative annotation

Candidate names are database-linked putative annotations and do not represent confirmed metabolite identities. Retention time, m/z, adduct type, molecular formula, database identifiers and ionization mode were obtained from the supplied annotated comparison table. Feature-level mass errors, MS/MS match scores, candidate-specific QC coefficients of variation, blank-sample evidence and authentic-standard confirmation could not be reliably linked to these candidate features in the supplied data package. ID, alignment identifier; RT, retention time; m/z, mass-to-charge ratio; HMDB, Human Metabolome Database; KEGG, Kyoto Encyclopedia of Genes and Genomes; Mode, ionization mode; Error, mass error; MS/MS score, tandem mass spectrometry spectral-match score; QC CV, coefficient of variation across pooled quality-control injections; Blank, blank-sample evidence; Std., authentic-standard confirmation; Status, identification status; NA, not available.

Supplementary Table S7. Quantitative QC repeatability metrics.

QC metric	All features (n = 1,105)	Positive mode (n = 633)	Negative mode (n = 472)
**Median CV (%)**	8.57	8.06	9.99
**CV IQR (%)**	5.44–14.80	5.33–13.15	5.82–16.92
**Features with CV ≤20%, n (%)**	937 (84.9%)	544 (85.9%)	394 (83.5%)
**Features with CV ≤30%, n (%)**	1,039 (94.0%)	589 (93.0%)	450 (95.3%)

CV was calculated as the sample standard deviation divided by the mean peak area across eight pooled-QC injections. The QC export was used only for descriptive run-level assessment and was not used to filter the fixed 959-feature model input. Candidate-specific QC CVs could not be reliably linked to the eight reported candidates and are therefore listed as NA in Supplementary Table S6.

Supplementary Table S8. Pairwise AUC comparisons with Random Forest.

Comparison	Pooled OOF AUC difference	95% CI	Raw P	BH-adjusted P
**RF − DT**	−0.005	−0.058 to 0.049	0.807	0.807
**RF − LR**	0.028	−0.053 to 0.124	0.580	0.773
**RF − XGBoost**	0.033	−0.010 to 0.086	0.146	0.292
**RF − SVM**	0.201	0.091 to 0.322	0.0010	0.0040

AUC differences were calculated from paired pooled out-of-fold predictions; positive values favor Random Forest. Confidence intervals and two-sided P values were estimated using 2,000 paired bootstrap replicates. Benjamini–Hochberg adjustment was applied across the four comparisons. Mean outer-fold AUC rankings and pooled out-of-fold AUC differences summarize different aspects of performance and may not have identical ordering. OOF, out-of-fold; BH, Benjamini–Hochberg; RF, Random Forest; DT, Decision Tree; LR, Logistic Regression; SVM, Support Vector Machine.

Supplementary Table S9. Exploratory clinical covariate-adjusted associations for the eight candidate features.

Candidate feature	Adjusted OR per 1-s.d. increase	95% CI	Adjusted P	BH-adjusted q
**Orotic Acid**	2.675×10^{-4}	5.230×10^{-9} to 2.160×10^{-2}	1.294×10^{-7}	1.035×10^{-6}
**Stigmasterol**	0.878	0.023 to 1.553	0.663	0.663
**Cocamidopropylbetaine**	0.611	0.128 to 1.457	0.317	0.362
**3-(Alpha,Alpha-Dimethylallyl) Psoralen**	0.703	0.388 to 1.164	0.166	0.222
**Isobutylamine**	0.484	1.383×10^{-15} to 0.959	0.0347	0.0694
**Alpha-Guaiaconic Acid**	0.422	0.158 to 0.855	0.0145	0.0386
**PFSM-carboxylic acid**	1.764	0.890 to 4.066	0.106	0.169
**3Alpha-Acetoxydihydrodeoxygedunin**	0.211	0.033 to 0.767	0.0144	0.0386

Associations were estimated using separate Firth-penalized logistic-regression models for each candidate feature, adjusted for age, sex, BMI, triglycerides, type 2 diabetes, gallstone history, cardiovascular history, smoking history, regular alcohol use, fatty liver and initial acute pancreatitis severity. Age, BMI and log-transformed triglycerides were standardized; odds ratios are reported per 1-s.d. increase in candidate-feature intensity. Analyses were restricted to complete cases (n = 57; 31 RAP events). P values were adjusted across the eight candidate features using the Benjamini–Hochberg procedure. This analysis was exploratory and post-selection; it was not considered independent validation or evidence of causality. CI, confidence interval; OR odds ratio; s.d., standard deviation; BH, Benjamini–Hochberg.

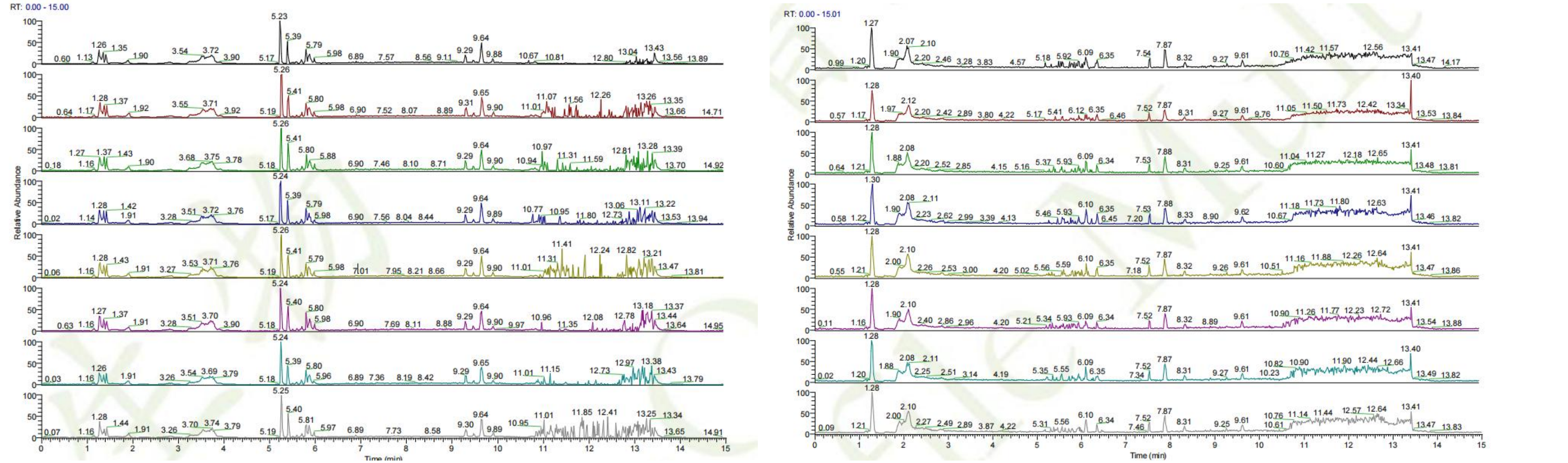

(a) Positive-ion mode base-peak chromatograms

(b) Negative-ion mode base-peak chromatograms

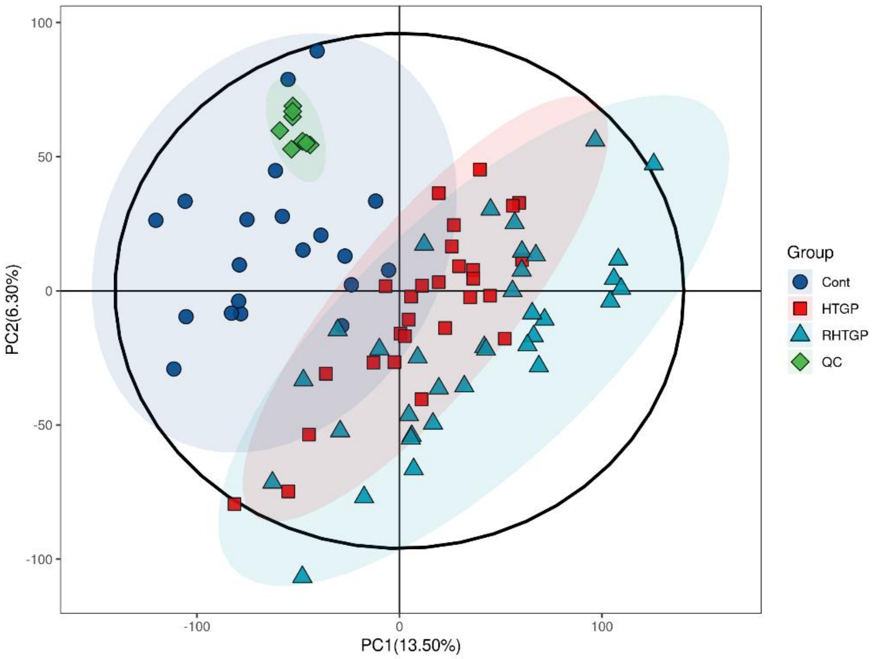

(c) PCA score plot in positive-ion mode

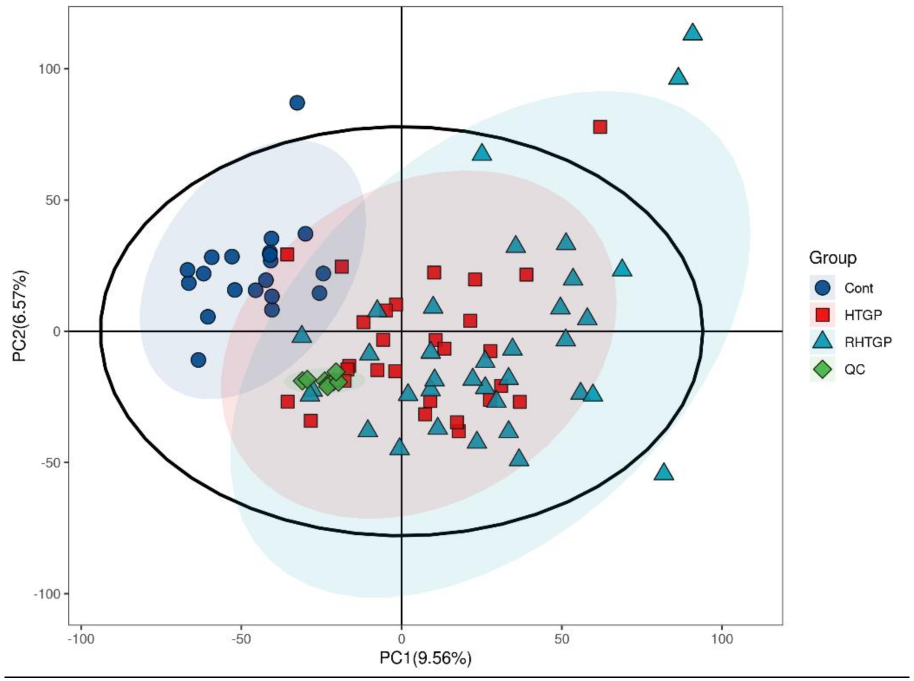

(d) PCA score plot in negative-ion mode

Supplementary Figure S1. Analytical quality-control assessment of the untargeted LC-MS/MS run.
(a,b) Base-peak chromatograms obtained from eight pooled-QC injections in positive- and negative-ion modes, respectively. (c,d) PCA score plots of the complete analytical dataset in positive- and negative-ion modes, respectively. The broadly comparable chromatographic profiles and compact clustering of QC samples provide qualitative evidence of analytical repeatability.

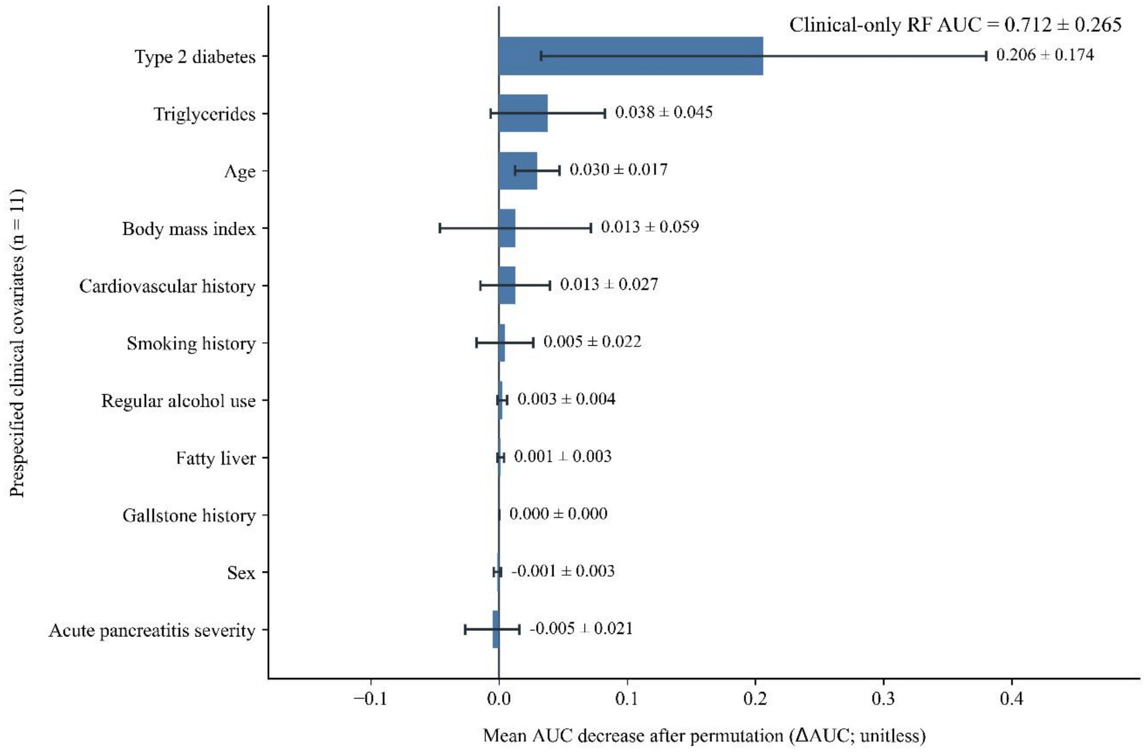

Supplementary Figure S2 Held-out permutation importance of prespecified clinical covariates. Permutation importance was evaluated using held-out data within each of the five outer cross-validation folds. Bars show the mean decrease in AUC after permuting each prespecified clinical covariate, and error bars denote the standard deviation across outer folds. The clinical-only random-forest model achieved a mean outer-fold AUC of 0.712 ± 0.265. Larger positive ΔAUC values indicate a greater contribution to held-out discrimination. Values close to zero or below zero indicate that permuting the covariate did not consistently reduce held-out AUC in this small cohort and should not be interpreted as evidence for a protective clinical association.
